# Structures and mechanism of human glycosyltransferase β1,3-*N*-acetylglucosaminyltransferase 2 (B3GNT2), an important player in immune homeostasis

**DOI:** 10.1074/jbc.RA120.015306

**Published:** 2020-11-22

**Authors:** Yue Hao, Amandine Créquer-Grandhomme, Noelle Javier, Aman Singh, Hao Chen, Paolo Manzanillo, Mei-Chu Lo, Xin Huang

**Affiliations:** 1 Department of Molecular Engineering, Amgen Research, Cambridge, Massachusetts, USA; 2 Amgen Postdoctoral Fellow Program, Amgen Research, Cambridge, Massachusetts, USA; 3 Department of Inflammation and Oncology, Amgen Research, South San Francisco, California, USA; 4 Department of Discovery Technologies, Amgen Research, South San Francisco, California, USA; 5 Department of Discovery Attribute Sciences, Amgen Research, South San Francisco, California, USA; 6 Department of Protein Technologies, Amgen Research, Cambridge, Massachusetts, USA

**Keywords:** glycobiology, poly-N-acetyl-lactosamine, glycosyltransferase, β1,3-N-acetylglucosaminyltransferase 2, structural biology, enzyme mechanism, B3GNT, *β*-1, 3-*N*-acetylglucosaminyltransferase, B3GT, β-1,3-glycosyltransferases, B4GALT, *β*-1, 4-galactosyltransferase, LacNAc, *N*-acetyl-lactosamine, SPR, surface plasmon resonance

## Abstract

β1,3-*N*-acetylglucosaminyltransferases (B3GNTs) are Golgi-resident glycosyltransferases involved in the biosynthesis of poly-*N*-acetyl-lactosamine chains. They catalyze the addition of the *N*-acetylglucosamine to the *N*-acetyl-lactosamine repeat as a key step of the chain elongation process. Poly-*N*-acetyl-lactosamine is involved in the immune system in many ways. Particularly, its long chain has been demonstrated to suppress excessive immune responses. Among the characterized B3GNTs, B3GNT2 is the major poly-*N*-acetyl-lactosamine synthase, and deletion of its coding gene dramatically reduced the cell surface poly-*N*-acetyl-lactosamine and led to hypersensitive and hyperresponsive immunocytes. Despite the extensive functional studies, no structural information is available to understand the molecular mechanism of B3GNT2, as well as other B3GNTs. Here we present the structural and kinetic studies of the human B3GNT2. Five crystal structures of B3GNT2 have been determined in the unliganded, donor substrate-bound, acceptor substrate-bound, and product(s)-bound states at resolutions ranging from 1.85 to 2.35 Å. Kinetic study shows that the transglycosylation reaction follows a sequential mechanism. Critical residues involved in recognition of both donor and acceptor substrates as well as catalysis are identified. Mutations of these invariant residues impair B3GNT2 activity in cell assays. Structural comparison with other glycosyltransferases such as mouse Fringe reveals a novel N-terminal helical domain of B3GNTs that may stabilize the catalytic domain and distinguish among different acceptor substrates.

Polylactosamine (poly-*N*-acetyl-lactosamine) is the essential feature of glycolipids and *N*-linked or *O*-linked glycans on glycoproteins. It contains multiple repeats of the *N*-acetyl-lactosamine (Gal*β*1-4GlcNAc) unit connected via *β*1-3 linkage and is frequently further decorated with other functional groups such as terminal sialic acids. Polylactosamine is involved in various biological processes including immune responses, through interacting with endogenous lectins or other carbohydrate-binding proteins. The multivalent interactions between the linear or modified polylactosamine and lectins create ordered lectin-saccharide lattices, which are proposed to initiate signal transduction and drive a series of cellular events including proliferation or death (1). For example, the ubiquitously expressed galectin-1, which recognizes a single repeat of *N*-acetyl-lactosamine (LacNAc)unit and functions as homodimer (2), binds T cell surface glycoproteins (CD3, CD7, CD43, and CD45) and induces redistribution of these proteins into segregated membrane microdomains, essential for triggering T cell apoptosis (3). The sialic acid–containing glycoconjugates could be recognized by Siglecs and contribute to inhibiting immune cells, maintaining immunological tolerance, modulating immune cell function, and regulating immune cell activation, maturation, and apoptosis (4).

The length of polylactosamine chain also affects immune reactions, and the long-chain polylactosamine on *N*-glycan is suggested to suppress excessive responses (5, 6, 7). The elongation of polylactosamine of bi-, tri-, and tetraantennary *N*-glycans (as well as *O*-glycans) involves two enzymes, β-1, 4-galactosyltransferase (B4GALT) and β-1, 3-*N*-acetylglucosaminyltransferase (B3GNT)(8, 9). They work in an alternating fashion to add galactose (Gal) and *N*-acetylglucosamine (GlcNAc) building blocks to the growing chain via *β*1-4 and *β*1-3 linkages (Fig. 1*A*). Seven B3GNTs (B3GNT2–B3GNT8), which belong to Glycosyl Transferase family 31 in the CAZy database, have so far been identified and functionally characterized *in vitro* using various oligosaccharide substrates with B3GNT2 demonstrating the strongest polylactosamine synthesizing activity (9). It takes uridine diphosphate (UDP)-activated GlcNAc as donor sugar and transfers GlcNAc to the Gal (acceptor sugar) in a divalent metal–dependent manner (10). The B3GNT2 gene is widely expressed in mouse with high-level expression detected only in the immune system (7). The subsequent *in vivo* study with B3GNT2 knockout mice showed dramatic reduction of polylactosamine on glycoproteins in tissues and cells of the immune system, despite the presence of the *N*-glycan core structures (5, 6). The immunocytes with diminished surface polylactosamine on *N*-glycan are hypersensitive and hyperresponsive to stimulation, indicating the inhibitory effect of polylactosamine on excessive immune responses and the crucial role of B3GNT2 as a major polylactosamine synthase (5, 6, 7). Supporting the connection of B3GNT2 and the immune system, genome-wide association studies revealed that single nucleotide polymorphisms reducing B3GNT2 expression are associated with autoimmune diseases including rheumatoid arthritis (11) in Japanese population and ankylosing spondylitis (12) and psoriasis (13) in people of European descent.

**Figure 1:**
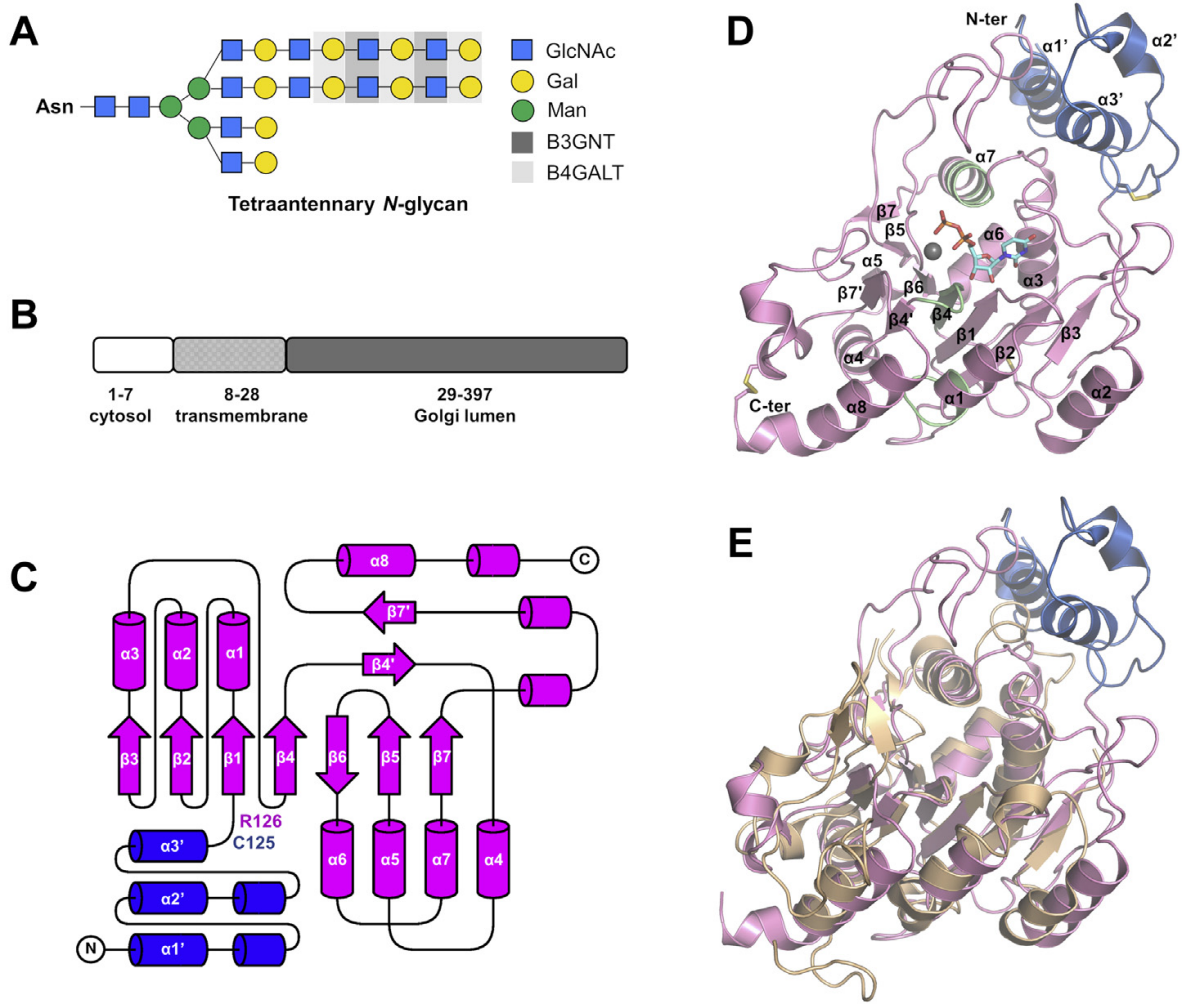
**Overall structure of human B3GNT2 luminal domain.***A,* synthesis of polylactosamine on tetraantennary *N*-glycan catalyzed by B3GNT and B4GALT enzymes. *B*, B3GNT2 is a type II transmembrane protein with a short N-terminal cytosolic segment, a transmembrane region, and a C-terminal domain in Golgi lumen. *C,* secondary structure topology of human B3GNT2 luminal domain. N-terminal domain is colored in *blue*, and GT-A domain is colored in *magenta*. Figure was prepared with TopDraw (48). *D*, overall structure of B3GNT2_UDP (chain A). N-terminal domain is colored in *blue*, and GT-A domain is colored in *pink*. The three common sequence motifs in B3GT family are colored in *green*. Magnesium ion is shown as a *gray sphere*, and UDP is shown as *cyan sticks*. Disulfide bonds are highlighted in *yellow*. *E*, superposition of human B3GNT2 luminal domain (*blue* and *pink*) and the catalytic domain (GT-A domain) of mouse Manic Fringe (*wheat*).

To obtain insights into the molecular mechanism of B3GNTs, we determined the crystal structures of the luminal domain of human B3GNT2 (Fig. 1*B*) at different stages of its enzymatic reaction: *apo* (2.35 Å), in complex with the donor substrate UDP-GlcNAc (2.26 Å), in complex with the acceptor substrate LacNAc (2.19 Å), in complex with the product UDP (1.85 Å), and in complex with both products UDP and trisaccharide GlcNAc*β*1-3Gal*β*1-4GlcNAc (2.20 Å), hereinafter referred to as B3GNT2_apo, B3GNT2_UDPGlcNAc, B3GNT2_LacNAc, B3GNT2_UDP, and B3GNT2_tri_UDP, respectively. We also demonstrated that B3GNT2 transglycosylation reaction proceeded through a sequential mechanism with kinetic analysis. Key residues in the catalytic domain essential for binding donor and acceptor substrates as well as its enzymatic activity were identified and further characterized by functional analysis of structure-based mutants. A novel helical domain N-terminal to the catalytic domain was observed and hypothesized to be involved in stabilization of the catalytic domain and differentiation of glycan acceptors.

## Results

### Overall structure of the luminal region of B3GNT2

The luminal domain structure of B3GNT2 was solved by single-wavelength anomalous dispersion phasing using an iodide derivative to a resolution of 2.50 Å. Subsequently, B3GNT2 structures in *apo* form or in complex with either substrates or products were determined by molecular replacement using the iodide derivative structure as the template. The six structures presented here were solved in different space groups and the number of molecules in the asymmetric unit also varied (Table S1). But a common dimeric complex formed by two molecules related with crystallographic symmetry was observed. The dimer interface is formed between helices α1, α2, α8 and loop β2-α2 from each molecule and buries a surface area of ∼1963 Å^2^ (Fig. S1*A*). Dimer formation was also confirmed in analytical size exclusion chromatography, with an estimated molecular weight of ∼84 kDa (Fig. S1, *B* and *C*).

The recombinant proteins used for crystallization contain residues Ser31–Cys397 or Lys45–Cys397, but only residues Pro55–Cys397 are well defined in the electron density maps, except for a disordered loop between residues Leu77 and Ser88.

The luminal domain of B3GNT2 comprises an N-terminal helical domain (Pro55–Cys125) and a C-terminal catalytic domain (Cys138–Cys397) as well as an extended linker (Arg126–Lys137) that connects the helical domain to the catalytic domain (Fig. 1, *C* and *D*). The helical domain consists of three helices α1’, α2’, and α3’ and is unique to B3GNT2 as no such domain has been observed for other glycosyltransferases. The catalytic domain adopts a mixed α/β fold commonly observed in glycosyltransferases of the GT-A superfamily (14) and is composed of a typical seven-stranded β sheet core (β1, β2, β3, β4, β5, β6, and β7) flanked mainly by α helices (α1, α2, α3, α4, α5, α6, and α7), a two stranded antiparallel β sheet formed by β4’ and β7’ (15), and an additional 12-residue α helix (α8) at the C terminus. The catalytic domain of human B3GNT2 closely resembles that of other glycosyltransferases, particularly mouse Fringe (PDB ID: 2J0B) (16) (Fig. 1*E*), with an RMSD of 2.17 Å for 181 Cα atoms despite 17% sequence identity between them. The main differences between B3GNT2 and Fringe lie in some of the loops. The α1-β2 loop and the β2-α2 loop are longer in B3GNT2 (12 and 12 residues, respectively) than in Fringe (7 and 2 residues, respectively), whereas the α6-α7 loop and the β7’-α8 loop are shorter in B3GNT2 (8 and 3 residues, respectively) than in Fringe (16 and 22 residues, respectively). The β3-α3 loop and the β5-β6 loop are ordered in the B3GNT2 structures, whereas they are disordered in the Fringe structure.

Three disulfide bonds are observed in B3GNT2: Cys96-Cys125 in the helical domain; Cys138-Cys235 and Cys367-Cys397 in the catalytic domain. In addition, glycans could be assigned for three glycosylation sites: Asn127 in the linker; Asn173 and Asn219 in the catalytic domain (Fig. S2*A*). The second GlcNAc ring of glycosylated Asn127 potentially forms hydrogen bonds with residues of the helical domain (Fig. S2*E*). Glycosylated Asn173 appears to be solvent exposed and the attached glycan has sufficient electron density only in certain chains in the B3GNT2 structures reported here. Glycosylated Asn219 is buried between the helical domain and the catalytic domain, with its first two GlcNAc rings stacked against Tyr122 of the helical domain and hydrogen bonded to both the helical domain and the catalytic domain (Fig. S2*E*).

### The N-terminal helical domain

**Figure 2:**
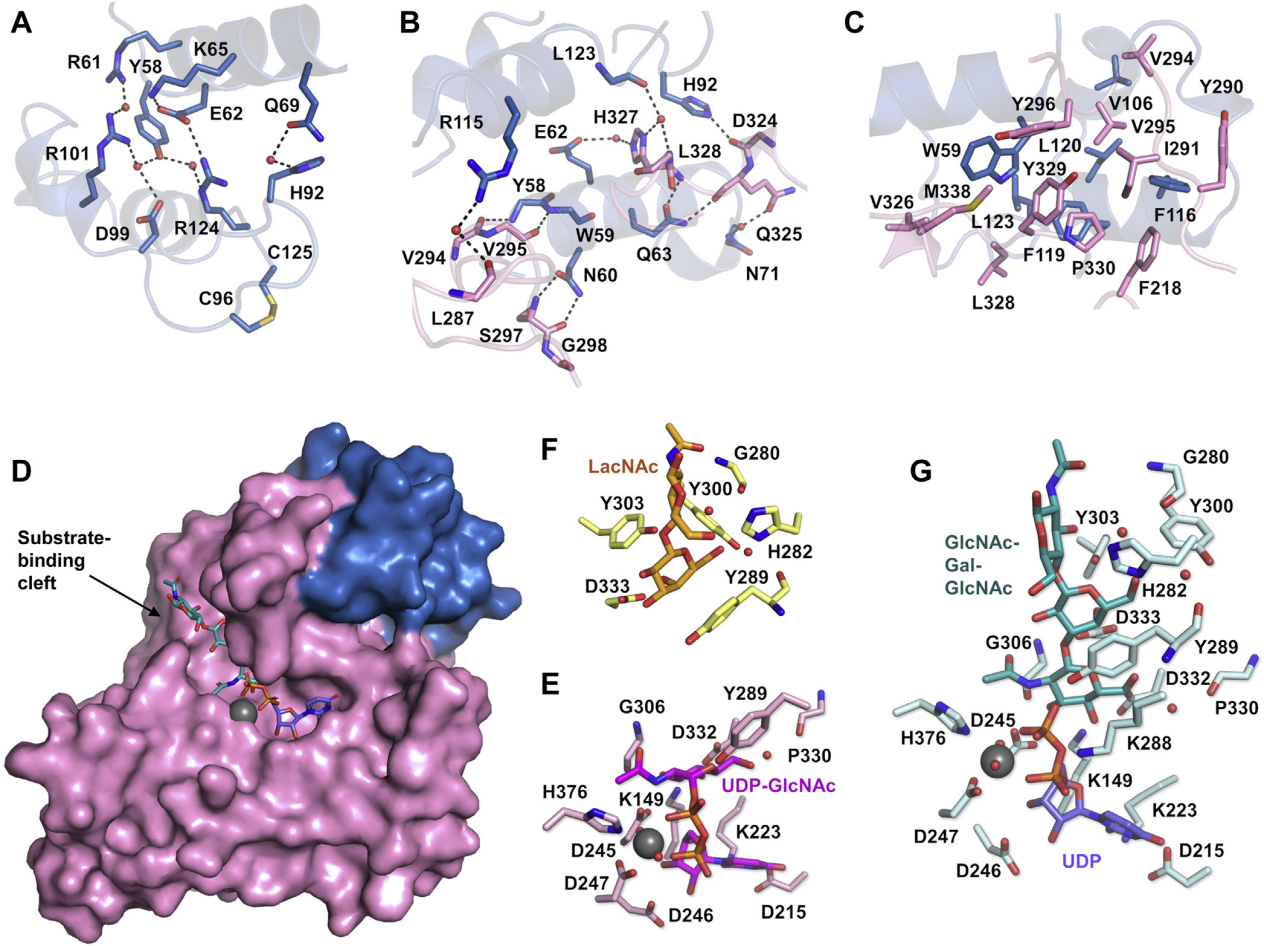
**N-terminal domain and substrate-binding cleft in GT-A domain.***A*, hydrogen-bonding interactions (*dashed lines*) within the N-terminal domain. Side chains of residues involved are shown as *sticks*. Water molecules are shown as *red spheres*. The Cys96-Cys125 disulfide bond is highlighted in *yellow*. *B,* hydrogen-bonding interactions (*dashed lines*) between the N-terminal domain (*blue*) and the GT-A domain (*pink*). Side chains and backbones of residues involved are shown as *sticks*. Water molecules are shown as *red spheres*. *C*, hydrophobic interactions between the N-terminal domain (*blue*) and the GT-A domain (*pink*). Side chains of residues involved are shown as *sticks*. *D*, surface representation of B3GNT2 structure, showing the substrate-binding cleft in GT-A domain. N-terminal domain is colored in *blue* and GT-A domain is colored in *pink*. Substrate-binding cleft is indicated with an *arrow*. GlcNAc*β*1-3Gal*β*1-4GlcNAc (*teal*) and UDP (*purple*), as well as magnesium ion (*gray sphere*), are shown in the substrate-binding cleft. *E*, donor substrate UDP-GlcNAc (*magenta*) is coordinated by residues (*pink*) in the substrate-binding cleft through hydrogen-bonding interactions (in structure B3GNT2_UDPGlcNAc). Magnesium ion is shown as a *gray sphere*, and water molecules are shown as *red spheres*. *F,* acceptor substrate LacNAc (*orange*) is coordinated by residues (*yellow*) in the substrate-binding cleft through hydrogen-bonding interactions (in structure B3GNT2_LacNAc). Water molecules are shown as *red spheres*. *G*, products GlcNAc*β*1-3Gal*β*1-4GlcNAc (*teal*) and UDP (*purple*) are coordinated by residues (*cyan*) in the substrate-binding cleft through hydrogen-bonding interactions (in structure B3GNT2_tri_UDP). Magnesium ion is shown as a *gray sphere*, and water molecules are shown as *red spheres*.

The N-terminal domain of luminal B3GNT2 adopts a loose helical bundle with three helices α1’, α2’, and α3’ (Fig. 1*D* and S3) and neither Dali nor PDBeFold search showed any result with significant structural similarity. α1’ and α3’ helices are parallel to each other, whereas the shortest α2’ helix is positioned above them at a 60° angle (Fig. S3). These three α helices are connected by two flexible loops each containing a one-turn 3_10_ helix. Although this three-helix bundle is not as tight as a typical three-helix bundle, there are some significant interactions within the helical domain, such as the hydrogen bonding network formed by Tyr58, Glu62, Lys65, Asp99, and Arg124. (Fig. 2*A*). In addition, the α1’-α2’ loop is covalently locked to the C terminus of the helical domain through Cys96-Cys125 disulfide bond (Fig. 2*A*).

The helical domain makes extensive contacts with the catalytic domain, primarily the β5-β6 loop and the α6-α7 loop, and together bury a surface area of over 2500 Å^2^, hydrophilic on one side and hydrophobic on the other. Residues from the α1’ helix (Tyr58, Trp59, and Asn60) form hydrogen bonds with the main chain nitrogens and oxygens of Val294, Val295, Ser297, and Gly298 from the β5-β6 loop (Fig. 2*B*). And residues from both the α1’ helix (Glu62 and Gln63) and loop regions (Asn71, His92, and Leu123) are involved in hydrogen bond interactions with residues from the α6-α7 loop (Asp324, Gln325, His327, and Leu328) (Fig. 2*B*). In addition, there are also extensive hydrophobic interactions between the helical domain and the catalytic domain including residues Trp59 (from the α1’ helix), Val106 (from the α2’-α3’ loop), Phe116, Phe119, Leu120, Leu123 (all four from the α3’ helix), and residues Tyr290, Ile291, Val294, Val295, Tyr296 (all five from the β5-β6 loop), Val326, Leu328, Tyr329, Pro330 (all four from the α6-α7 loop) (Fig. 2*C*).

### The substrate-binding cleft

By soaking B3GNT2 crystals with either the donor substrate UDP-GlcNAc or the acceptor substrate LacNAc, we were able to obtain B3GNT2 structures (B3GNT2_UDPGlcNAc and B3GNT2_LacNAc) in the substrate-bound states. The substrate-binding cleft is located on one end of the core β sheet of the catalytic domain (Figs. 1*D* and 2*D*).

In the B3GNT2_UDPGlcNAc structure, the donor substrate UDP-GlcNAc is positioned at the bottom of the cleft with the GlcNAc moiety close to the center of the cleft (Figure 1, Figure 2*A* and 2*E*). The side chains of Asp215 and Lys223 are hydrogen bonded to the uracil ring of the UDP moiety, whereas the side chain of Asp246 is hydrogen bonded to the ribose hydroxyls. The pyrophosphate group of UDP clamps the divalent Mg ion in a bidentate manner. Two pyrophosphate oxygen atoms, Asp247 from the signature DXD motif (Asp245-Asp246-Asp247 in B3GNT2) (17), a conserved His376, and two water molecules, coordinate the Mg ion with the tetragonal bipyramidal geometry. The side chains of Lys149, Asp245 (the other Asp from the signature DXD motif), Tyr289, and Asp332 are involved in hydrogen bond interactions with the GlcNAc moiety with Asp245 engaged in additional interaction with the nitrogen of the *N*-acetyl group. These interactions force the GlcNAc moiety to bend toward the cleft wall, almost perpendicular to the pyrophosphate and parallel to the uracil ring, and fully expose the C1 atom for the nucleophilic attack.

The acceptor substrate LacNAc (Gal*β*1-4GlcNAc) is bound to the top of the cleft and more solvent exposed (Fig. S4*B* and 2*F*). The Gal ring for the anticipated glycosidic bond formation reaction is located close to the center of the cleft, whereas the GlcNAc ring is close to the solvent. The hydroxyls of the Gal ring are engaged in direct hydrogen bond interactions with the side chains of His282 and Asp333 and water-mediated hydrogen bond interaction with the side chain of Tyr300. The Gal ring is also stacked against the side chain of Tyr289. In contrast, the GlcNAc ring is only involved in a water-mediated hydrogen bond with the main chain oxygen of Gly280 through one of its hydroxyls.

### Binding kinetics of B3GNT2

**Figure 3:**
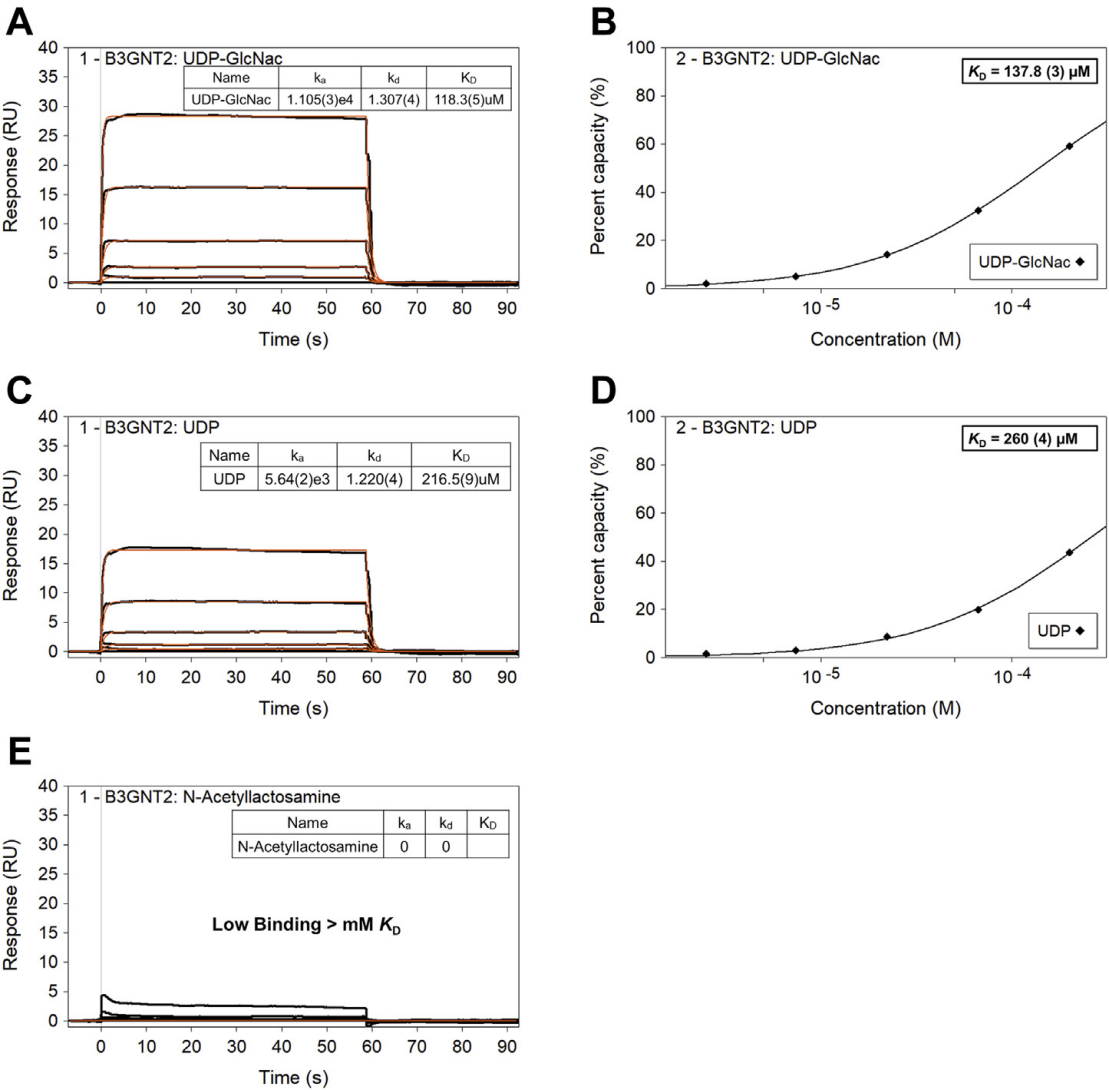
**SPR binding characterization of UDP-GlcNAc, UDP, and LacNAc**. Steady-state (equilibrium) affinity binding data of UDP-GlcNAc (*B*) and UDP (*D*) to B3GNT2 coupled on SA sensor. The kinetic binding model fit (*A* and *C*) shows 1:1 binding of test analytes with rapid equilibrium and fast dissociation.

Biophysical binding interactions of the substrates (UDP-GlcNAc and LacNAc) and the product (UDP) to B3GNT2 coupled to streptavidin sensor were measured using surface plasmon resonance (SPR). A 1:1 binding was assumed based on the crystal structures, which clearly reveal the stoichiometry of the reaction. The steady-state (equilibrium) binding affinities (*K*_D_) of UDP-GlcNAc and UDP were calculated to be ∼140 μM (Fig. 3*B*) and ∼260 μM (Fig. 3*D*), respectively, whereas the binding affinity of LacNAc could not be measured reliably (likely millimolar *K*_D_) owing to very weak binding signal (Fig. 3*E*). The simple 1:1 kinetic model fitted (Fig. 3*A* and *C*) double referenced binding data of UDP-GlcNAc and UDP suggested weak binding affinity with B3GNT2 (partial ligand occupancy with nonsaturation up till 200 μM top concentration with rapid equilibrium and fast dissociation), a profile characteristic of small molecule binders with weak (in the micromolar range) affinity.

### Kinetic mechanism of B3GNT2

**Figure 4:**
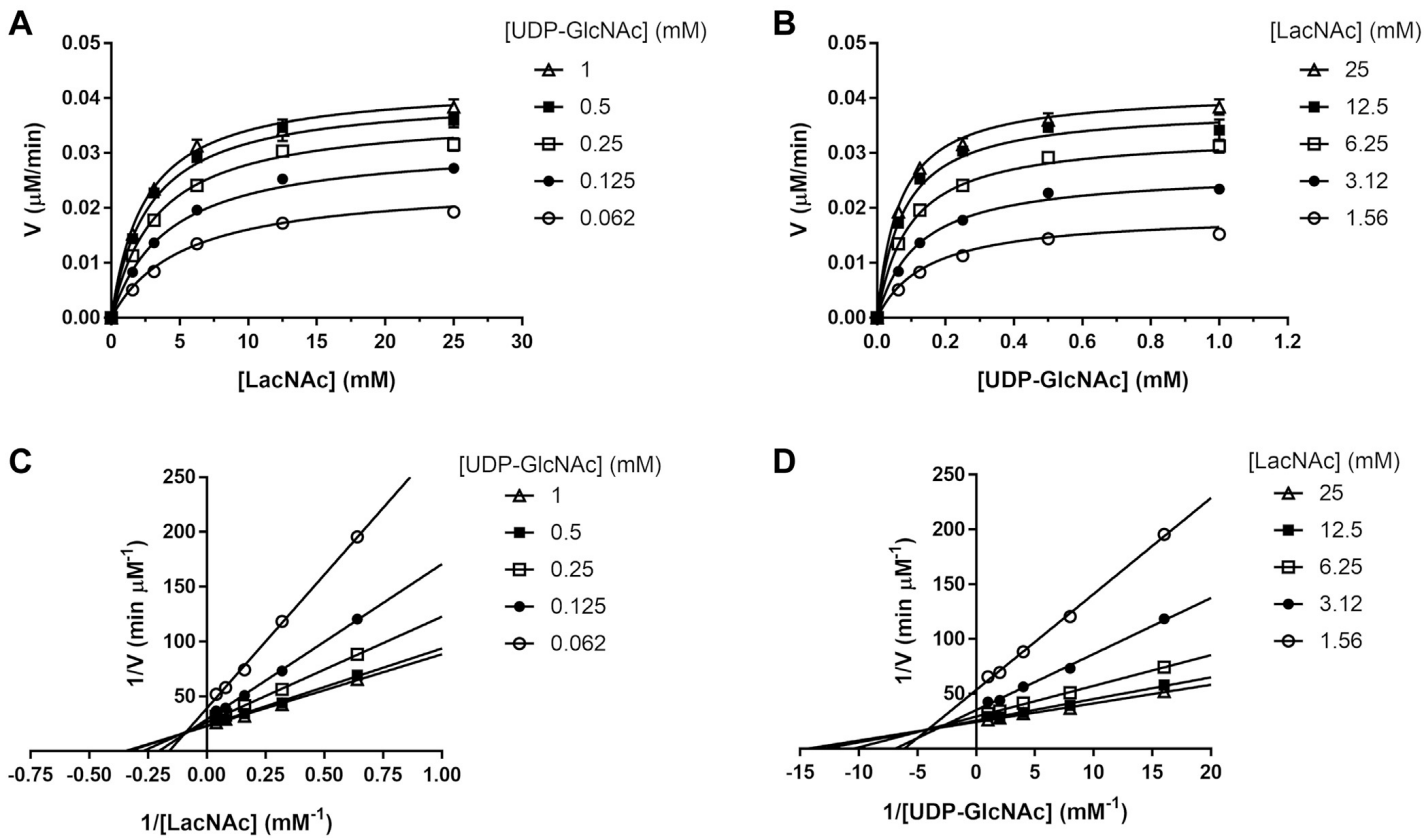
**Steady-state kinetic experiment for B3GNT2-catalyzed transglycosylation.***A*, Michaelis–Menten plots of the initial velocities versus LacNAc concentrations for each fixed concentration of UDP-GlcNAc. The data set was globally fit to Equation 1 that describes the rapid-equilibrium random-order sequential mechanism, and the best fit is represented by the solid lines. *B*, Michaelis–Menten plots of the initial velocities versus UDP-GlcNAc concentrations for each fixed concentration of LacNAc. The data set was globally fit to Equation 1 that describes the rapid-equilibrium random-order sequential mechanism, and the best fit is represented by the solid lines. *C*, double-reciprocal plots of initial velocities versus LacNAc concentrations for each fixed concentration of UDP-GlcNAc. The *solid lines* show the linear regression fit. *D*, double-reciprocal plots of initial velocities versus UDP-GlcNAc concentrations for each fixed concentration of LacNAc. The *solid lines* show the linear regression fit.

Two-substrate reactions catalyzed by enzymes can proceed through either a sequential mechanism or a ping-pong mechanism (18). In a sequential mechanism, both substrates must bind to the enzyme to form the ternary complex before catalysis can occur. In contrast, in a ping-pong mechanism, one product must be released before the other substrate can react. To determine the kinetic mechanism of B3GNT2, we measured the initial velocities at various concentrations of UDP-GlcNAc and LacNAc using the UDP-Glo assay (Fig. 4, *A* and *B*) and constructed the double-reciprocal plots of the initial velocities versus the substrate concentrations (Fig. 4, *C* and *D*). The lines in the double-reciprocal plots intersect, indicating that B3GNT2 utilizes a sequential mechanism rather than a Ping-Pong mechanism. In addition, the pattern of the intersecting lines in the double-reciprocal plots excludes a rapid-equilibrium compulsory-order sequential mechanism because this mechanism requires the lines in the double-reciprocal plot to intersect on the y-axis. Since we were able to obtain the structures of the binary enzyme–substrate complexes for both UDP-GlcNAc and LacNAc and SPR data showed fast-on and fast-off binding kinetics for both substrates, we fit the initial velocities at various substrate concentrations to the rapid-equilibrium random-order sequential mechanism (Equation 1). The kinetic parameters obtained from the fit are: *K*_UDP-GlcNAc_ = 216 ± 36 μM, *K*_LacNAc_ = 8.99 ± 1.75 mM, α = 0.24 ± 0.06, and *k*_cat_ = 35.8 ± 0.7 min^−1^. The equilibrium dissociation constants of the binary enzyme–substrate complex obtained from kinetic analysis (216 μM for UDP-GlcNAc and 8.99 mM for LacNAc) are similar to the values measured by the SPR study. The cooperativity factor of α (0.24) obtained from the fit indicates that the two substrates have positive binding cooperativity and they are held about fourfold more tightly in the ternary complex. The catalytic constant (*k*_cat_) of B3GNT2 (35.8 min^−1^) is within the range reported for other glycosyltransferases, which typically have low turnover numbers (19).

### Reaction mechanism

**Figure 5:**
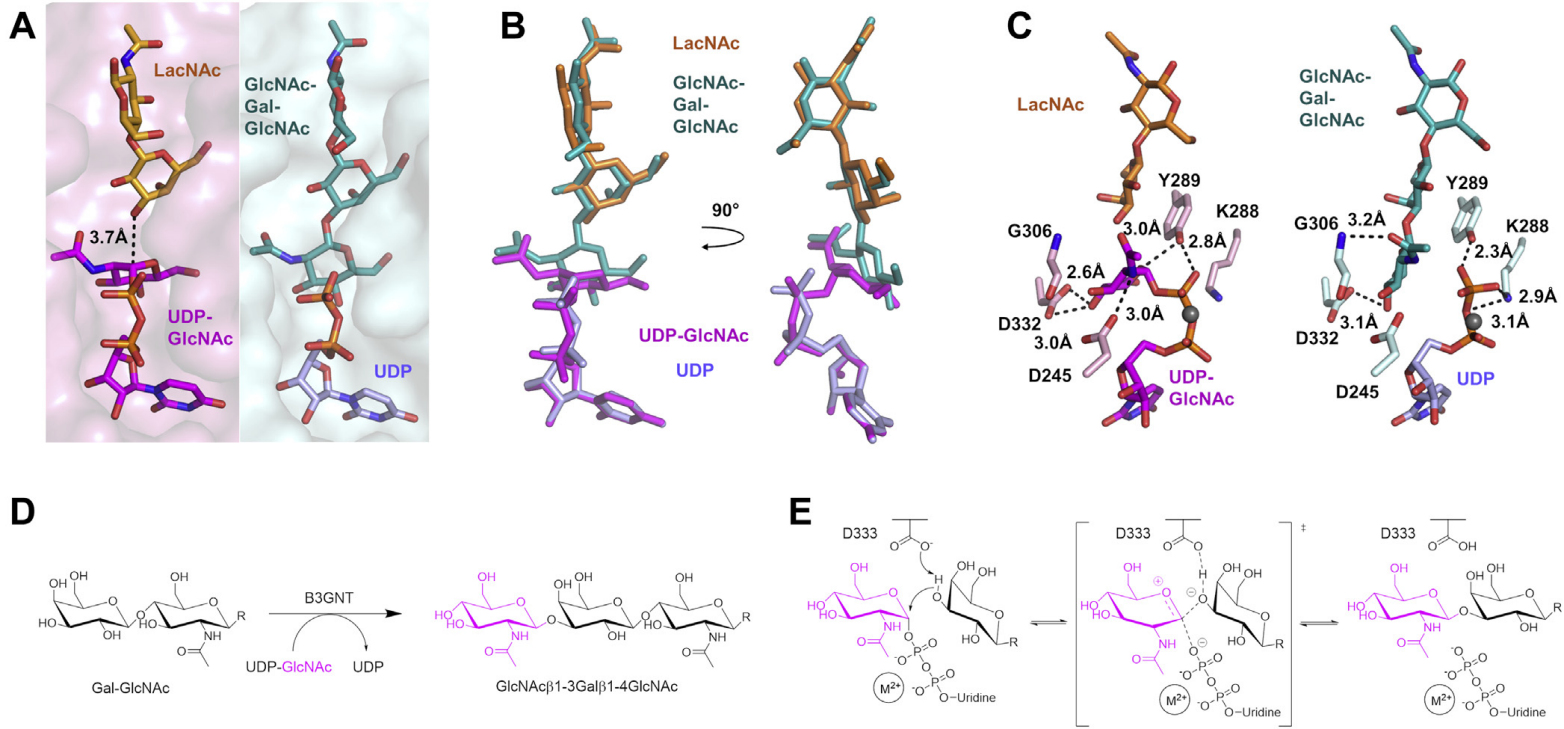
**Mechanism of B3GNT2 glycosyltransferase reaction.** (*A*) *Left panel*, superposition of B3GNT2_UDPGlcNAc and B3GNT2_LacNAc at the substrate-binding cleft. The surface representation of substrate-binding cleft of B3GNT2_UDPGlcNAc is shown in *pink*. The distance between the C1 of the GlcNAc in UDP-GlcNAc (*magenta*) and the hydroxyl group on C3 of the Gal in LacNAc (*orange*) is marked by *dashed line*. *Right panel*, the substrate-binding cleft (*cyan surface*) in B3GNT2_tri_UDP is shown in the same orientation as the left panel and the two products (GlcNAc*β*1-3Gal*β*1-4GlcNAc in *teal* and UDP in *purple*) are shown as *sticks*. (*B*) Superposition of the substrates and products in the substrate-binding cleft. UDP-GlcNAc, *magenta*; LacNAc, *orange*; GlcNAc*β*1-3Gal*β*1-4GlcNAc, *teal*; UDP, *purple*. (*C*) Change of the hydrogen-bonding interactions in the substrate-binding cleft before (*left panel*) and after (*right panel*) the reaction takes place. UDP-GlcNAc, *magenta*; LacNAc, *orange*; GlcNAc*β*1-3Gal*β*1-4GlcNAc, *teal*; UDP, *purple*; magnesium ion, *gray sphere*. (*D*) Glycosyltransferase reaction catalyzed by B3GNT2. (*E*) Mechanism of a divalent-metal-dependent inverting glycosyltransferase reaction. Asp333 is proposed to be the active site base. The GlcNAc to be transferred is proposed to undergo an oxocarbenium ion-like transition state.

A sequential mechanism of the B3GNT2 reaction implies that both substrates can bind the enzyme at the same time. And superposition of the B3GNT2_UDPGlcNAc and B3GNT2_LacNAc structures shows that the acceptor substrate LacNAc binds right above the donor substrate UDP-GlcNAc in the substrate-binding cleft. The hydroxyl group on C3 of the Gal in the acceptor substrate points to the C1 of the GlcNAc in the donor substrate at a distance of 3.7 Å (Fig. 5*A*). To obtain a ternary complex structure, we soaked B3GNT2 crystals with both donor and acceptor substrates as well as magnesium ion at the same time. Interestingly, electron density was clearly observed for both products UDP and trisaccharide GlcNAc*β*1-3Gal*β*1-4GlcNAc but no remaining density for the residual substrates UDP-GlcNAc or LacNAc, indicating that the glycosyltransferase reaction took place during soaking and the products were not yet released. As a result, we were able to obtain the crystal structure of B3GNT2 with both products bound (B3GNT2_tri_UDP) (Fig. S4*C* and 2*G*).

Structural comparison of the product-bound and the substrate-bound B3GNT2 reveals a clear picture of the inverting glycosyltransferase reaction. The Asp333, which is 2.7 Å from the hydroxyl group on C3 of Gal in the acceptor substrate LacNAc, serves as the active site base and abstracts the proton from this hydroxyl group (Fig. 2*F*). This facilitates the nucleophilic attack on the exposed C1 of the GlcNAc in the donor substrate UDP-GlcNAc, which undergoes an oxocarbenium ion–like transition state (17) (Fig. 5*E*). Owing to arrangement of the two substrates in the active site, the nucleophilic attack occurs at the *si* face of the transition state oxocarbenium ion, while the UDP leaves from the *re* face. Consequently, the C-P bond breaks in UDP-GlcNAc and the new β-1,3-glysosidic bond forms between GlcNAc and Gal, accompanied by GlcNAc converting from α anomer to β anomer (Fig. 5*B*). The GlcNAc ring therefore rotates by 30°, losing interactions with Tyr289 but establishing new hydrogen bond with the backbone nitrogen of Gly306 through its *N*-acetyl group (Fig. 5*C*). The pyrophosphate of UDP is also able to rotate slightly to release the torsion and be further stabilized by Tyr289 and Lys288 (Fig. 5*C*). Notably, all the other residues coordinating the two products are the same residues recognizing the two substrates and they undergo minimal conformational changes during the reaction.

To capture the conformations of the enzyme during the entire reaction cycle, we also determined two more structures of B3GNT2: unliganded and bound to only UDP (for the state when GlcNAc*β*1-3Gal*β*1-4GlcNAc is released) (Fig. S4*D*). Comparison of the unliganded structure to the substrate-bound or the product-bound structure demonstrates that the substrate-binding cleft of *apo* B3GNT2 is available to accommodate substrates for the reaction to occur. Nevertheless, some subtle conformational changes in the cleft have been observed at different stages of the reaction cycle (Fig. S7). One example is the catalytic residue Asp333 aforementioned. The side chain of Asp333 points away in *apo* B3GNT2 (Fig. S7, *A* and *B*) but rotates to engage the substrates upon binding of either the donor or the acceptor substrate (Fig. S7, *C* and *D*). It stays engaged after the reaction (Fig. S7*E*) and only to disengage after the release of the product GlcNAc*β*1-3Gal*β*1-4GlcNAc (Fig. S7*F*).

### B3GNT2 active site mutations impair polylactosamine synthesis

**Figure 6:**
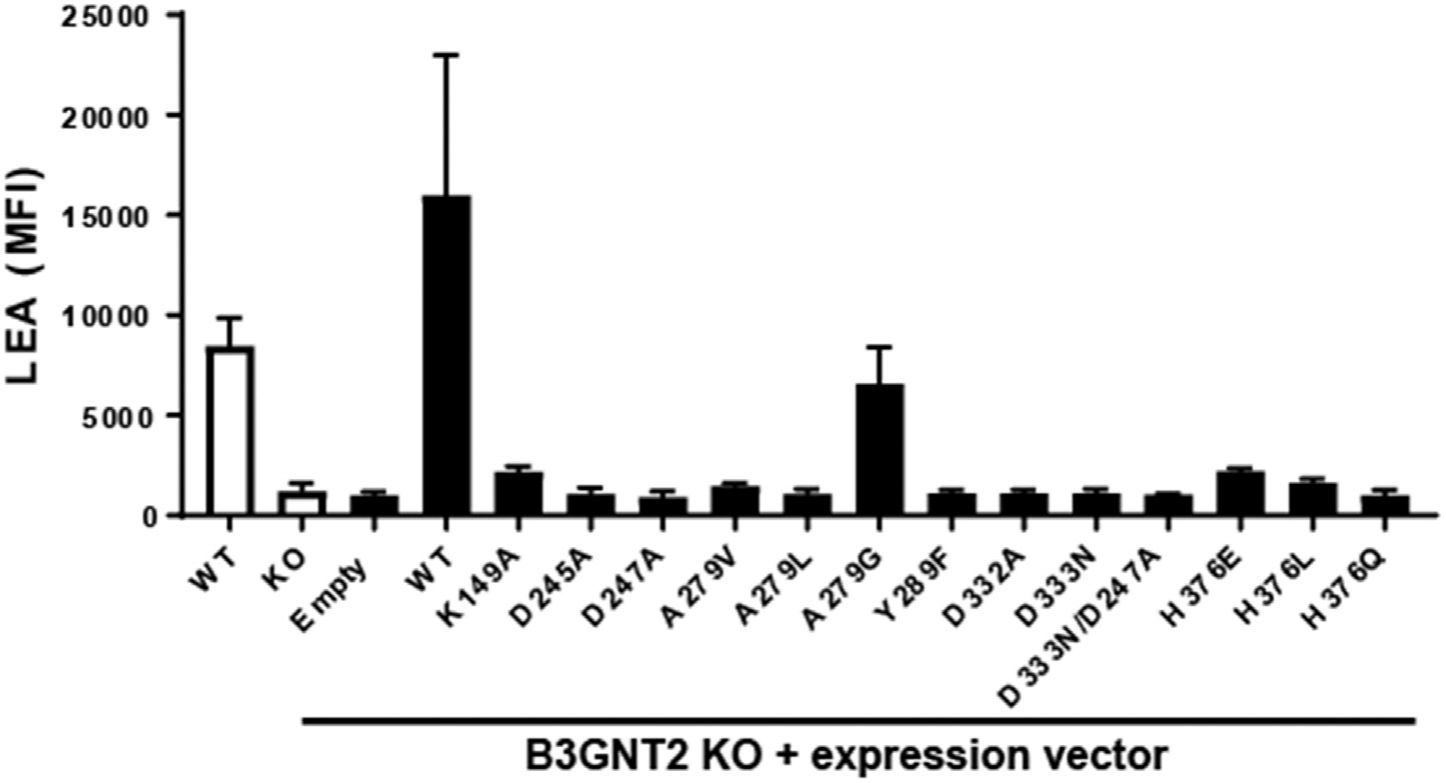
**Assessment of B3GNT2 point mutants.** Flow cytometry analysis of LEA lectin binding was performed on WT, B3GNT2 KO, or B3GNT2 KO cells expressing the indicated B3GNT2 point mutation. Data shown are the average cell surface LEA mean fluorescence intensity (MFI) from two independent experiments.

To evaluate the importance of specific residues in catalysis and substrate recognition, we reconstituted B3GNT2-deficient Jurkat cells with various point mutants and assessed polylactosamine levels via flow cytometry using the LEA lectin derived from *Lycopersicon esculentum*. In agreement with previous reports (5, 6), CRISPR-mediated deletion of B3GNT2 in Jurkat cells resulted in a significant decrease in LEA binding relative to wildtype (WT) cells (Fig. 6) demonstrating B3GNT2 as the major *N*-acetylglucosaminyltransferase involved in polylactosamine biosynthesis. LEA staining in B3GNT2 KO cells could be recovered by retroviral overexpression of WT B3GNT2 but not empty GFP vector alone. In contrast, reconstitution of KO cells with point mutations within residues required for metal binding (D247A, H376Q, H376L, H376E), substrate binding (K149A, D245A, Y289F, D332A), or within the active site base (D333N) failed to restore cell surface polylactosamine levels, suggesting significantly reduced enzyme activity (Fig. 6). Ala279 from the β5-β6 loop is less than 4 Å from the acetyl group of the GlcNAc of the acceptor substrate (Fig. S8). A mutation of Ala279 to either Val or Leu may create steric hindrance with the GlcNAc (Fig. S8) and failed to restore LEA staining when expressed in KO cells (Fig. 5). In comparison, expression of a A279G point mutant partially restored polylactosomaine levels, although not as robust as WT B3GNT2. Ala279 is conserved among the seven B3GNTs (B3GNT2-B3GNT8) (Fig. S9), except for B3GNT3 and B3GNT6 where it is replaced by a Val and a Ser respectively.

## Discussion

B3GNTs are Golgi-resident glycosyltransferases with a short seven-residue N-terminal cytosolic segment, a transmembrane helix, and a large luminal domain, which consists a stem region and the catalytic domain. Sequence alignment of homologous glycosyltransferases often displays a well-conserved catalytic domain and highly dissimilar stem region varying in both amino acid composition and length (20). Most eukaryotic glycosyltransferases structures published so far contain only the catalytic domain, since the stem region is considered as having little secondary organization and its presence potentially hampers successful crystallization (20). The B3GNT2 proteins used in our crystallization trials comprise both the catalytic domain and the stem region, and our structures show that the stem region is a helical domain consisting of three helices α1’, α2’, and α3’. There are hydrogen bond interactions between the secondary elements as well as the Cys96-Cys125 disulfide bond within the helical domain. The α2’-α3’ loop and the α3’ helix are highly conserved in other B3GNTs, whereas the two disulfide cysteines are completely conserved in other B3GNTs except for B3GNT5 (Fig. S9), suggesting that other B3GNTs likely contain similar helical domain in their stem regions. The *N*-glycosylation in the stem region was also thought to contribute to local conformation (20, 21), but the two predicted glycosylation sites (Asn79 and Asn89) in the α1’-α2’ loop of B3GNT2 were disordered in our structures suggesting no structural contribution from the *N*-glycosylation in the stem region. Furthermore, these two glycosylation sites are also not conserved in other B3GNTs.

There are several hypotheses on the functions of the stem region of Golgi glycosyltransferases. The kin recognition model suggests that the stem region is involved in glycosyltransferase localization and retention in Golgi apparatus and in oligomerization of enzymes in the same particular compartment (22, 23), as exemplified by the critical roles of the stem regions of β-1,2-*N*-acetylglucosaminyltransferase I (NAGT-I) (24) and β1,6-*N*-acetylglucosaminyltransferase V (GnT-V) (25). A potential benefit of colocalization and oligomerization of the glycosyltransferases, which act in succession in glycan synthesis, is that it may improve efficiency of the process, by conveniently channeling the intermediates from the product position to the acceptor substrate position along the transfer step (20, 26). However, in the study of β1,4-galactoside α2,6-sialyltransferase I (ST6GalI), the authors showed the stem region seems to participate in glycan acceptor recognition, probably by restricting the access to the acceptor subsite to accommodate selective glycans, through interacting with the catalytic domain (21). In addition, it was proposed that the stem region of B4GalT1 (β-1,4-galactosyltransferase) serves as a chaperone to enhance the correct folding of the catalytic domain (27, 28).

Our structures reported here demonstrate no significant interactions between the stem regions of B3GNT2 molecules in the crystal lattice. It is not very likely for the stem region of B3GNTs to oligomerize and locate these enzymes to Golgi. The stem region of B3GNT2 is a helical domain and has extensive interactions with the β5-β6 loop and the α6-α7 loop of the catalytic domain, resulting in a large interface between these two domains. Consequently, the β5-β6 loop is ordered in the B3GNT2 structures and forms a major part of the substrate binding cleft. In comparison, the β5-β6 loop is disordered in the Fringe structure and a significant part of the substrate binding cleft is missing. In addition, glycosylated Asn219 is buried between the two domains and this glycosylation site is the only one (of five) conserved among all B3GNTs. All these would indicate that one of the functions of the stem region of B3GNTs is to stabilize the catalytic domain for the enzymatic activity. It was previously reported that the glycosyltransferases from the same subfamily are capable of constructing the same sugar linkage but discriminating among glycan acceptors, probably enabled by some structural features (21). The function to distinguish different glycan acceptors is likely carried out by the more variable stem region rather than the highly conserved catalytic domain of B3GNTs. Understanding the extent of recognition of glycan acceptors by the stem region of B3GNTs awaits structural studies of their complexes. We would also like to suggest it is possible for the stem region of B3GNTs to form complex with a B4GALT enzyme to efficiently accomplish the sequential addition of GlcNAc and Gal to the polylactosamine chain. Future structural studies will be needed to test this hypothesis.

Sequence alignment of all seven B3GNTs (B3GNT2–B3GNT8) (Fig. S9) showed the catalytic domain is highly conserved. Three common sequence motifs have been identified in the gene family of β-1,3-glycosyltransferases (B3GT), including B3GALT, B3GalNAcT, and B3GNT groups. They are (I/L)RxxWG, (F/Y)(V/L/M)xxxDxD, and (E/D)D(A/V)(Y/F)xGxC/S (9, 29). In B3GNT2 structure, all three motifs are located in the catalytic domain (Fig. 1*D*). Motif 1 (Ile161-Arg162-X_2_-Trp165-Gly166) is located at the α1 helix, contributing to the formation of the core structure. Motif 2 (Phe240-Val241-X_3_-Asp245-X-Asp247) spans the whole β4 strand and the β4-β4’ loop with the DxD sequence coordinating divalent metal, together with the pyrophosphate group of the donor substrate. Motif 3 (Asp332-Asp333-Val334-Tyr335-X-Gly337-X-Cys339) spans the majority of the α7 helix, with Asp332 in contact with the donor sugar and Asp333 acting as the active site base for catalysis.

Among the seven B3GNTs, B3GNT2, B3GNT3, B3GNT4, and B3GNT8 are involved in the elongation of polylactosamine chain in *N*-linked or *O*-linked glycosylation (9). Sequence alignment of the four B3GNTs showed that the active site base Asp333 and other residues (except His 282) directly involved in substrate recognition are conserved among B3GNT2, B3GNT3 and B3GNT4. However, some of these residues are less conserved in B3GNT8, with Asp245 and Tyr289 in B3GNT2 replaced by Gln and Phe, respectively, providing less coordination to the substrates. In addition, His376 for divalent metal coordination is substituted in B3GNT8 with a Arg. This could explain why B3GNT8 is 20-fold less active than B3GNT2 (30).

Aberrant expression and function of B3GNT2 have been reported to be linked to various human diseases. Besides the aforementioned autoimmune diseases, recent study on genetic mutations related to glycosylation pathway in colorectal cancer also demonstrated that B3GNT2 mutations are enriched in tumor tissues and patient-derived cell lines (31). The three mutations identified (R6X, P186T, and D247H) significantly alter B3GNT2 subcellular location or impair enzymatic activity and consequently enhance the migratory potential of cancer cells, observed in biochemical and cell assays (31). Except the R6X mutation, which generates a soluble form of B3GNT2 through an alternative translation start, the other two mutations are located in the catalytic domain based on the structures presented here. The active site mutation D247H may severely impact proper metal coordination and the catalysis, clearly demonstrated in the activity assay with either LacNAc-PNP or Lactose-PNP as substrate (31). Pro186 is located at the β2-α2 loop and is not conserved among the B3GNTs. It is over 10 Å away from the substrate-binding cleft and exposed to solvent. It is not clear from our B3GNT2 structures how this mutation could influence enzyme activity or cell function.

In summary, we report the structures of human B3GNT2 luminal domain, including both the catalytic domain and the stem region that is a novel helical domain, and reveal the reaction mechanism of transglycosylation by B3GNT2. Essential residues in substrate binding and catalysis are identified, and the glycosyltransferase reaction mechanism is proposed. We also hypothesize that the helical domain stabilizes the catalytic domain and possibly distinguishes among different glycan acceptors.

## Experimental procedures

### Expression and purification of B3GNT2

The expression construct of human B3GNT2 (UniProt Q9NY97) luminal domain (Ser31–Cys397 or Lys45–Cys397) was designed with an N-terminal gp64 secretion signal peptide and a cleavable N-terminal 8×His tag. The whole segment was cloned into pORB vector with the Golden Gate assembly method (32). The resulting plasmid was cotransfected with linearized baculovirus genomic DNA (AB Vector, LLC) into *sf9* cells and high-titer recombinant baculovirus stock was then generated according to manufacturer’s instructions. *sf9* cells with a density of 2.5 × 10^6^ cells/ml in serum-free medium were infected with P2 virus at a multiplicity of infection of 5. Spent medium was harvested 65 h post infection by centrifugation at 2000*g* for 15 min.

Stock solutions of 1 M Tris-HCl pH 8.0, 1 M CaCl_2_, and 1 M NiCl_2_ were added to medium to final concentration of 50 mM, 1.25 mM, and 1 mM to adjust pH and neutralize metal chelating agents. Filtered medium was concentrated by tangential flow filtration and incubated overnight with Ni-NTA resin pre-equilibrated with 20 mM Hepes-NaOH pH 7.5, 250 mM NaCl (buffer A). Impurities were removed by washing the resin with buffer A supplemented with 40 mM imidazole and B3GNT2 was eluted with buffer A containing 250 mM imidazole. B3GNT2 fractions were dialyzed against buffer A overnight, and meanwhile, 8×His tag was cleaved by TEV protease. Preliminarily purified protein was concentrated and subject to size exclusion chromatography with Superdex 200 10/300 column (GE Healthcare) in buffer 20 mM Hepes-NaOH pH 7.5, 150 mM NaCl (buffer C for B3GNT2 Ser31-Cys397) or buffer 20 mM Hepes-NaOH pH 7.5, 250 mM NaCl, 50 mM imidazole, 1 mM MgCl_2_, 0.5 mM DTT (buffer D for B3GNT2 Lys45-Cys397). To calculate the molecular weight standard curve, the following proteins (Bio-Rad Laboratories, Inc) were used: bovine thyroglobulin (670 kDa), bovine γ-globulin (158 kDa), chicken ovalbumin (44 kDa), horse myoglobin (17 kDa), and vitamin B12 (1.35 kDa).

### Crystallization and structure determination

B3GNT2 purified from gel filtration was concentrated to 5.7 mg/ml (B3GNT2 Ser31–Cys397) or 7.7 mg/ml (B3GNT2 Lys45–Cys397). Initial crystallization screening was set up with or without 0.5 mM UDP using the sitting-drop vapor diffusion method with commercial sparse matrix screens at 4 °C. Diffraction-quality crystals appeared in condition 24% PEG1500, 20% glycerol with rod shape. Optimization of crystals with or without 0.5 mM UDP was carried out with hanging-drop vapor diffusion method in 24-well trays (Hampton Research). The mother liquor was used as a cryoprotectant solution before being flash frozen in liquid nitrogen.

To obtain iodide-derived crystal, a large B3GNT2 Ser31–Cys397 crystal (incubated with 0.5 mM UDP and 1 mM MgCl_2_, prior to crystallization) was soaked in soaking solution (24% PEG1500, 20% glycerol) supplemented with 0.5 M NaI for 30 s at room temperature. Single-wavelength anomalous dispersion data were collected at a wavelength of 1.85 Å at Advanced Light Source (Berkeley, CA, USA) beamline 5.0.2 using a Pilatus 3 6M detector. A total of 1440 diffraction images were collected with 0.5 ° oscillation angle. Data were indexed and integrated with *XDS* (33) and scaled with Aimless (34, 35, 36) in CCP4 suite (37). Autosol (38, 39, 40, 41) in Phenix suite was used for locating the 42 iodide atoms, phasing, density modification, and initial model building. A relatively complete structure was built using Autobuild (42) in Phenix suite. Iterative manual adjustment in COOT (43) and refinement of the model using Phenix.refine (44) were carried out to achieve the final structure. The density of UDP is only strong enough in chain A and chain C in the asymmetric unit.

Except the structure of the iodide derivative, all other structures of B3GNT2 were determined with B3GNT2 Lys45-Cys397 crystals. Diffraction data of B3GNT2_apo and B3GNT2_UDP were collected directly using crystals from screening trays or optimization trays. To obtain B3GNT2_UDPGlcNAc crystals, *apo* crystals were soaked in soaking solution with 10 mM UDP-GlcNAc and 10 mM MgCl_2_ for 2 h at 4 °C. LacNAc soaking was performed using B3GNT2 crystals cocrystallized with UDP, with 10 mM LacNAc in soaking solution (no UDP) for 1 h. During structure determination, some electron density possibly from the uracil ring could be observed but it was insufficient to build the full UDP. Therefore, only the LacNAc molecule was built in the B3GNT2_LacNAc structure. To obtain B3GNT2_tri_UDP crystals, *apo* crystals were soaked in soaking solution with 10 mM UDP-GlcNAc, 10 mM LacNAc, and 10 mM MgCl_2_ for 30 min at 4 °C. Glycosyltransferase reaction occurred during soaking, and products GlcNAc*β*1-3Gal*β*1-4GlcNAc and UDP were observed in the substrate-binding cleft.

Diffraction data of the *apo* or ligand-bound crystals were collected at a wavelength of 1.00 Å, either at Advanced Photon Source (Lemont, IL, USA) beamline 22-ID (SER-CAT) using a MARMOSAIC 300 CCD detector or at Advanced Light Source (Berkeley, CA, USA) beamline 5.0.2 using a Pilatus 3 6M detector. Datasets were either indexed, integrated, and scaled with HKL2000 (45) or indexed and integrated with *XDS* (33) and scaled with Aimless (34, 35, 36) in CCP4 suite (37). The structure of iodide-derivatized B3GNT2 was used as template for molecular replacement using Phaser (40). The relatively complete structures were built using Autobuild (42) in Phenix suite and ligands were modeled in subsequently. Ligand restraints were generated with eLBOW (46). Iterative manual adjustment in COOT (43) and refinement using Phenix.refine (44) were carried out to achieve the final structures. Refinement process was monitored with MolProbity (47). The data collection and refinement statistics were summarized in Table S1. In structures B3GNT2_UDPGlcNAc, B3GNT2_LacNAc, and B3GNT2_UDP, the electron density of substrate/product ligands in the two molecules of the asymmetric unit was comparable, and chain A’s were used to generate figures. In structure B3GNT2_tri_UDP, the solvent-exposed GlcNAc showed increased B factors compared with the rest of the atoms in product GlcNAc*β*1-3Gal*β*1-4GlcNAc.

### Surface plasmon resonance assay for binding characterization

The label-free SPR was used to probe direct interactions of test analytes (UDP-GlcNAc, UDP, LacNAc) to biotinylated B3GNT2, which was coupled to high-affinity streptavidin (S series SA)-carboxymethylated dextran sensor chip on Biacore T200 bioanalyzer (GE Healthcare) with running buffer (50 mM Tris-HCl pH 8.0, 200 mM NaCl, 1 mM MnCl_2_, 0.013% Triton X-100, 3% DMSO (v/v)) at 25 °C. The SA sensor was normalized (using 70% glycerol) and conditioned with 5 × 1-min injections of SA conditioning buffer (50 mM NaOH in 1 M NaCl). Biotinylated B3GNT2, 100 nM, in coupling buffer (50 mM Hepes-NaOH, pH 8.0, 150 mM NaCl, 0.01% Tween20) was flown over flow cell at 10 μl/min till surface density of 4500 RU (theoretical R_max_ 30–50 RU) was achieved. The flow cell with ligand (B3GNT2) and mock surface (no protein) was capped off with a 5-min injection of 50 μM D-Biotin in coupling buffer and system baselines were allowed to stabilize overnight. The test analytes were prepared as 100 mM stock in water and later diluted into running buffer while maintaining the final DMSO concentration at 3% (v/v). Test analytes (and positive controls) were sequentially injected (in triplicate) over SA sensor as 5 point, 1:3 dilution dose response series (2.5–200 μM), at 30 μl/min for 1 min of association and 2 min of dissociation time. The raw data (sensograms) were analyzed using Scrubber 2.0 (Biologic Software, Australia). All test analyte raw binding response data was Y normalized, cropped, and applied with solvent correction curves, double referenced (subtraction of nearest blank buffer injection signal to minimize bulk refractive shifts) and processed data were fitted to the steady state affinity model (1:1 monovalent binding) while allowing maximum binding signal to fit freely along with floating bulk RI (refractive index). The double-referenced raw data were also fitted to nonlinear regression-based 1:1 kinetic binding model with single (global) R_max_ to determine kinetic rate constants. Owing to rapid equilibrium and fast dissociation of test analytes steady state affinity data were selected (over kinetic affinity) to report binding affinity (*K*_D_) along with standard error of fitness of equilibrium dissociation constant (*K*_D_, μM) to model.

### *In vitro* UDP-Glo assay for B3GNT2 activity

To investigate B3GNT2 kinetic mechanism, the initial velocities were measured at various combinations of substrate concentrations using the UDP-Glo assay (Promega, Madison, WI, USA), which detects the production of UDP from the B3GNT2 reaction. The assay was conducted in a 384-well white OptiPlate (PerkinElmer, Shelton, CT, USA) in a total volume of 40 μl. The B3GNT2 enzyme reaction (in 20 μl) contained 1.25 nM B3GNT2 (Lys29–Cys397) and varying concentrations of UDP-GlcNAc (1, 0.5, 0.25, 0.125, 0.062 mM) and LacNAc (25, 12.5, 6.25, 3.12, 1.56 mM) in a reaction buffer of 50 mM Tris-HCl pH 7.5, 2.5 mM MnCl_2_, 0.01% Tween20, and 0.0125% bovine serum albumin. The reactions were initiated by addition of B3GNT2 at different times and then were terminated simultaneously by addition of an equal volume (20 μl) of UDP Detection Reagent. Plates were incubated at room temperature for 1 h, and luminescence was measured on an Envision multimode reader (PerkinElmer). The initial velocities were determined from the slopes of the progress curves in the linear range and the luminescence units were converted to the UDP concentrations based on the standard curve of UDP. The kinetic parameters were obtained by fitting the initial velocities to Equation 1, which describes the rapid-equilibrium random-order sequential mechanism (18), using GraphPad Prism (GraphPad, San Diego, CA, USA).

(1) $$V\;=\;\frac{k_{cat}\left[E\right]\left[A\right]\left[B\right]}{\alpha K_{A}K_{B}\;+\;\alpha K_{A}\left[B\right]\;+\;\alpha K_{B}\left[A\right]\;+\;\left[A\right]\left[B\right]}$$

where *V* is the initial velocity at given [A] and [B], *k*_cat_ is the catalytic constant or the turnover number, [E] is the enzyme concentration, *K*_A_ and *K*_B_ are equilibrium dissociated constants for the binary enzyme–substrate complexes EA and EB, respectively, and *α* is the cooperativity factor that expresses how the binding of one substrate affects the affinity of enzyme for the other substrate.

### B3GNT2 gene deletion in Jurkat cells

The B3GNT2 gene was deleted in Jurkat cells using an electroporation-based CRISPR-CAS9 system. Briefly, Alt-R reagents from Integrated DNA Technologies were used for CRISPR-Cas9–mediated gene deletion according to manufacturer’s protocols. TracrRNA/crRNA duplexes and RNP complexes were prepared and electroporation of the RNP complex in Jurkat cells was performed using the Lonza P3 Primary Cell 4D-Nucleofector X Kit (V4XP-3032). *B3GNT2*-specific crRNA CGGTTCCAGTATGCCTCGGG was designed with the website crispor.tefor.net/. B3GNT2 deletion was assessed by evaluating loss of LEA (lectin from *L. esculentum*) binding by flow cytometry 9 days post electroporation. Low-LEA-binding (*i.e.*, B3GNT2-deficient) cells were sorted as a bulk population on a BD FACSMelody cell sorter.

### B3GNT2 mutagenesis and transduction of *B3GNT2*-deficient Jurkat cells

Mutagenesis was carried out on the B3GNT2 (Myc-DDK-tagged) pCMV6-Entry vector purchased from Origene (catalog #RC208100) with the Agilent Quik Change II XL Site-Directed Mutagenesis kit. Primers used for mutagenesis are listed in Table S2. The mutated tagged B3GNT2 sequences were subsequently cloned into the MSCV-IRES-GFP (pMIG) retroviral vector (Addgene #20672), using *Xho*I as a restriction cloning site, allowing for coexpression of B3GNT2 and GFP.

Retroviral particles were prepared via cotransfecting the pMIG vectors with the pAmpho vector, which expresses the 4070A amphotropic envelope protein under the control of the CMV immediate-early promoter (Clontech #PT3750-5), and a gag/pol expressing vector (Cellbiolabs #RV-111) in HEK293T cells. Lentiviral particles were obtained from concentrating culture supernatants of transfected HEK293T cells with the Retro-X Concentrator (Clontech #631456). B3GNT2-deficient Jurkat cells (0.5 × 10^6^ cells per reaction) were spin infected (1 h at 32 °C, 2500 rpm) with the different pMIG vectors in 7 μg/ml Polybrene.

### Flow cytometry analysis of LEA lectin binding

Lectin binding was assessed several days post infection. Cells were stained for 20 min at 4 °C with 1 μg/ml biotin-conjugated lectin from *L. esculentum* (tomato) (LEA) (Sigma #L0651) in FACS buffer (PBS, 0.5 mM EDTA, 1% FBS). Cells were subsequently washed in FACS buffer and incubated for 15 min at 4 °C with 0.125 μg/ml APC-conjugated Streptavidin (Biolegend #405207). Finally, after a second wash in FACS buffer, cells were incubated for 15 min at 4 °C with Efluor 506 fixable viability dye (eBioscience #65-0866-14) (1/2000 dilution), washed in FACS buffer and resuspended in 200 μl FACS buffer and acquired on a BD LSR-II flow cytometer. Streptavidin only (no lectin) was used as a negative control for LEA binding.

### Data availability

The structures presented here have been deposited in the Protein Data Bank (PDB) with accession codes of 7JHI, 7JHK, 7JHL, 7JHM, 7JHN, and 7JHO. All other data are included in this manuscript.

## Conflict of interest

The authors declare that they have no conflicts of interest with the contents of this article.
