# Supplementary material for: Structures and mechanism of human glycosyltransferase β1,3-N-acetylglucosaminyltransferase 2 (B3GNT2), an important player in immune homeostasis
Source: J Biol Chem. 2020 Nov 22;296:100042. doi: 10.1074/jbc.RA120.015306 (PMC7948737; doi:10.1074/jbc.RA120.015306)
Supplement: Supplementary data [file mmc1.pdf]

Supporting Information

Structures and mechanism of human glycosyltransferase β1,3-*N*-acetylglucosaminyltransferase 2 (B3GNT2), an important player in immune homeostasis

Yue Hao[1,2]*, Amandine Créquer-Grandhomme [3], Noelle Javier[4], Aman Singh[5], Hao Chen[6], Paolo Manzanillo[3], Mei-Chu Lo[4], Xin Huang[1]*

[1]Department of Molecular Engineering, Amgen Research, 360 Binney Street, Cambridge, MA 02142, USA

[2]Amgen Postdoctoral Fellow Program, Amgen Research, 360 Binney Street, Cambridge, MA 02142, USA

[3]Department of Inflammation and Oncology, Amgen Research, 1120 Veteran Boulevard, South San Francisco, CA 94080, USA

[4]Department of Discovery Technologies, Amgen Research, 1120 Veteran Boulevard, South San Francisco, CA 94080, USA

[5]Department of Discovery Attribute Sciences, Amgen Research, 1120 Veteran Boulevard, South San Francisco, CA 94080, USA

[6]Department of Protein Technologies, Amgen Research, 360 Binney Street, Cambridge, MA 02142, USA

*Corresponding authors: Yue Hao, Xin Huang

yhao@amgen.com, xinhuang_us@yahoo.com

The supporting information includes:

Figures S1 to S9

Tables S1 to S2

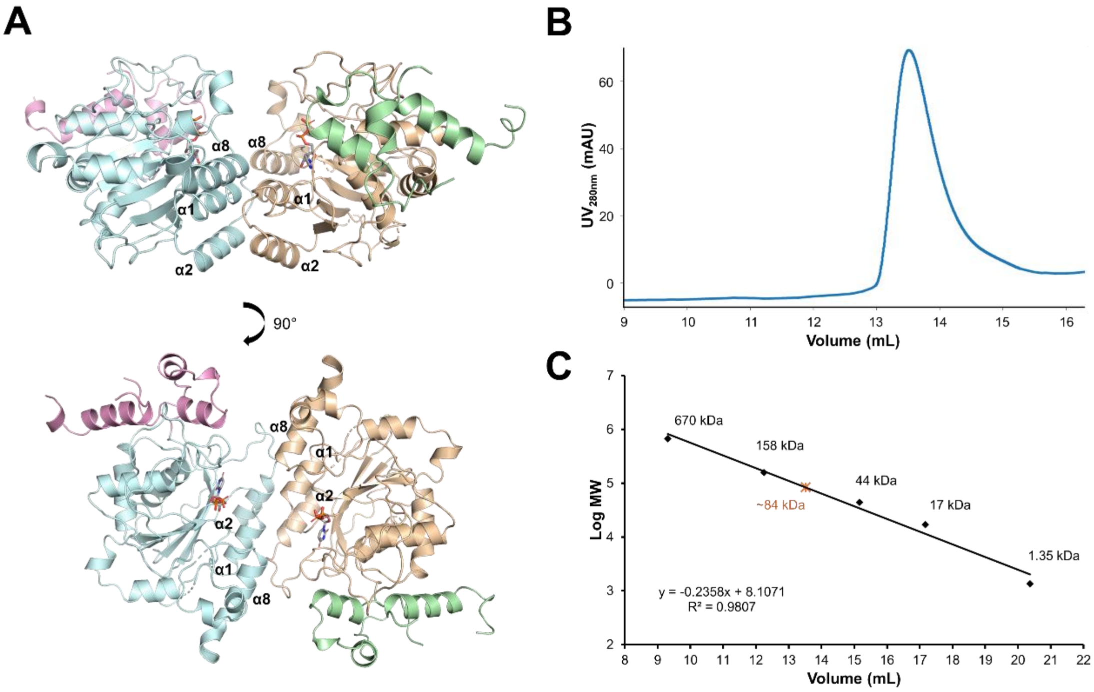

Fig. S1 Dimer formation of B3GNT2. (A) Dimeric complex of B3GNT2 observed in structure. One protomer of B3GNT2_UDP (chain A) is colored in pink and cyan. The symmetry-related protomer is colored in green and wheat. UDP is shown as grey sticks. The dimer interface is formed between helices α1, α2, α8 and loop β2-α2 from each protomer. **(B)** Analytical size exclusion chromatography of B3GNT2 (Lys45-Cys397) without His tag. **(C)** Molecular weight standard curve with the molecular weight of each standard indicated. The molecular weight calculated (~84 kDa) closely agrees with a dimeric form of B3GNT2 (Lys45-Cys397) monomer (~41 kDa).

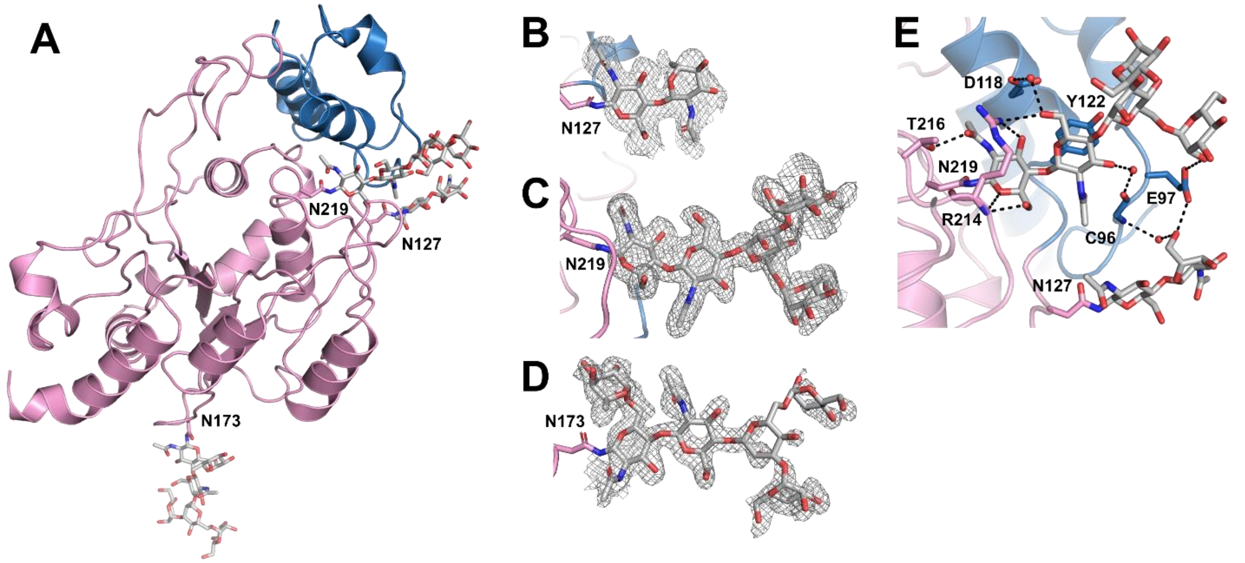

Fig. S2 Glycosylation sites of B3GNT2. (A) Overall structure of B3GNT2_UDP (chain A) with glycans displayed. N-terminal domain is in blue and catalytic domain is in pink. *N*-glycans are shown as grey sticks. Polder maps of the *N*-glycans attached to N127 **(B)**, N219 **(C)** and N173 **(D)** are contoured at σ=3.0. **(E)** Interactions between the *N*-glycans (grey sticks) on Asn127 and Asn219 and residues from N-terminal domain (blue) and catalytic domain (pink) in B3GNT2_UDP.

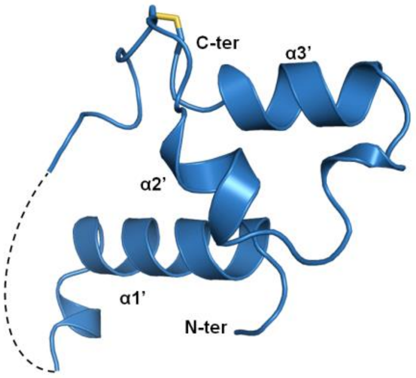

Fig. S3 Structure of the N-terminal domain (Pro55-Cys125). The missing loop is represented by dashed line. The Cys96-Cys125 disulfide bond is highlighted in yellow.

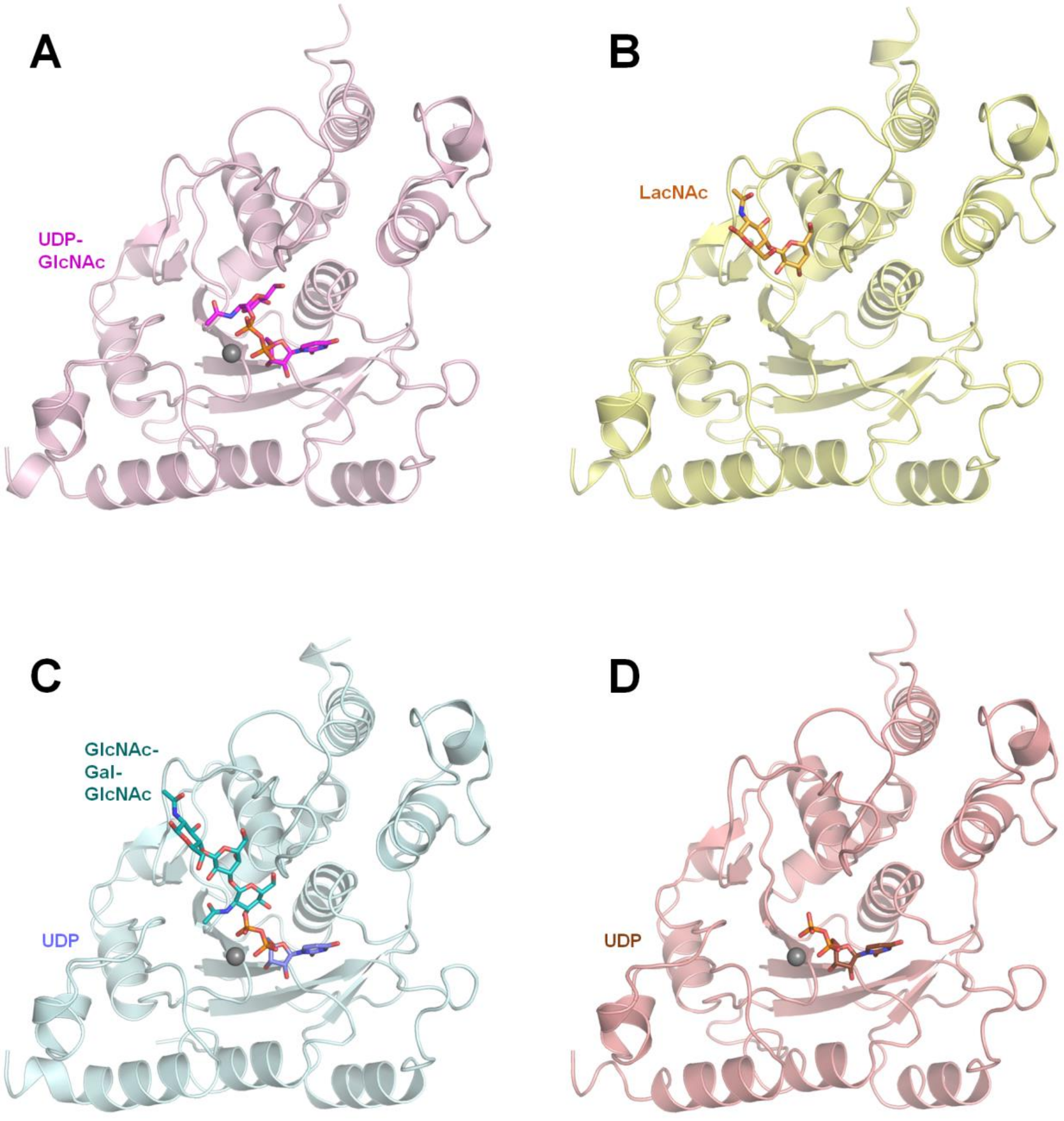

Fig. S4 Overall structures of B3GNT2_UDPGlcNAc (chain A) (A), B3GNT2_LacNAc (chain A) (B), B3GNT2_tri_UDP (C), and B3GNT2_UDP (chain A) (D).

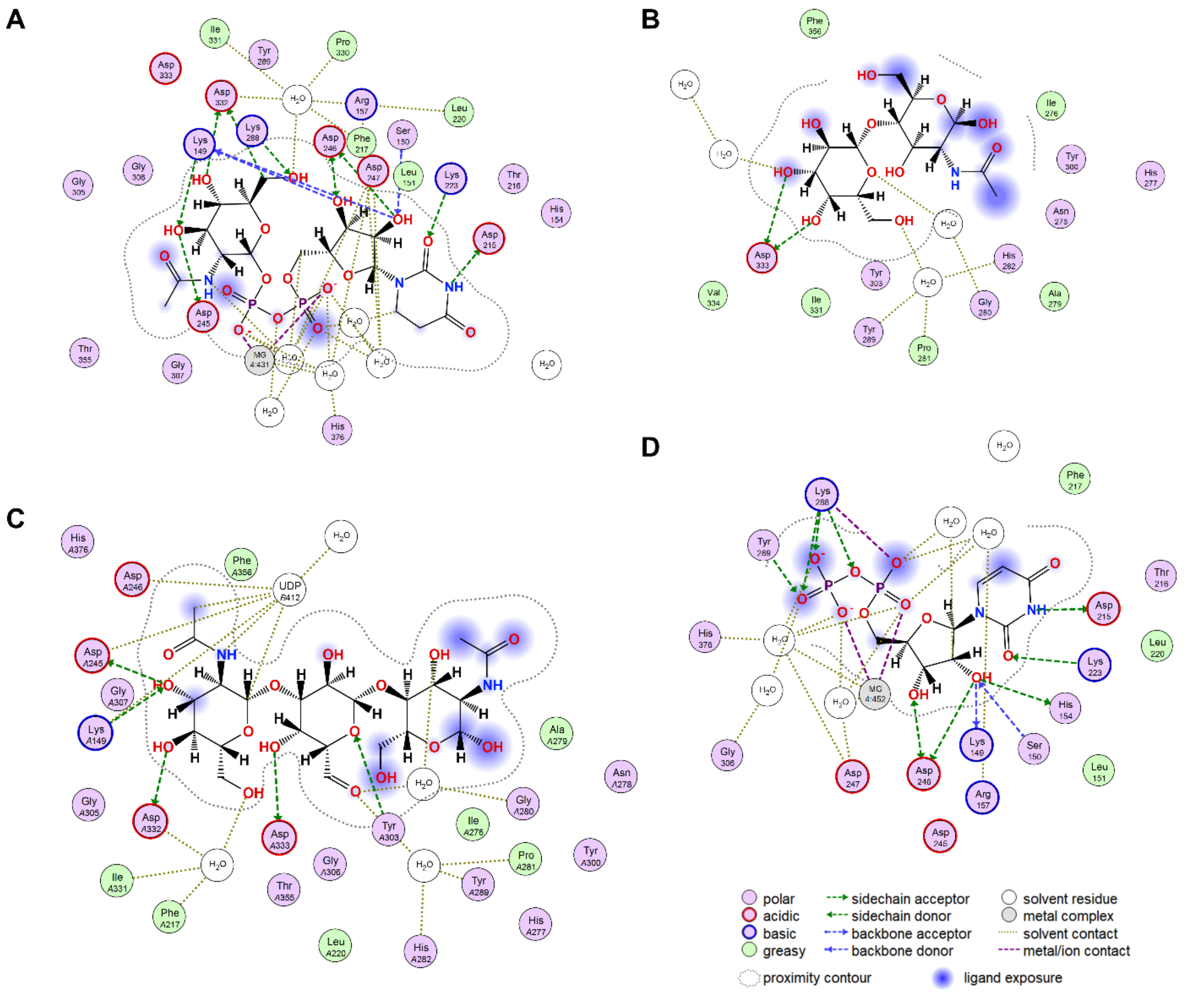

Fig. S5 Coordination of substrate or product in the substrate-binding cleft. 2D diagrams generated in MOE (Chemical Computing Group) show the interactions between the substrate-binding cleft and UDP-GlcNAc **(A)**, or LacNAc **(B)**, or GlcNAcβ1-3Galβ1-4GlcNAc and UDP **(C)**, or UDP **(D)**. Residues and solvent molecules within 4.5 Å from the ligands are displayed.

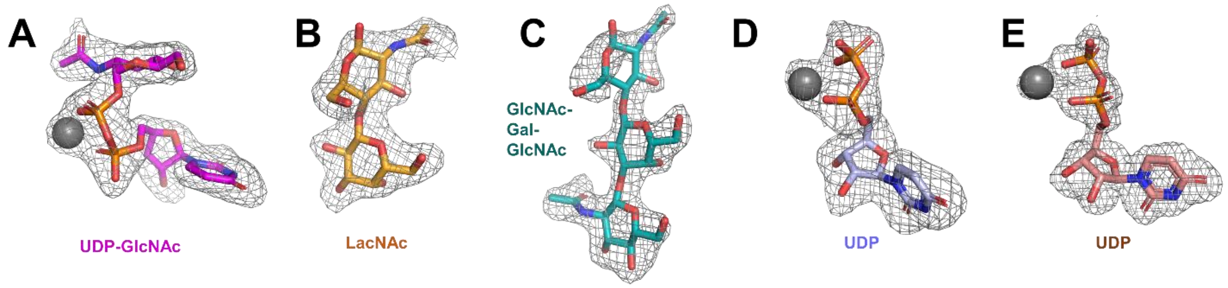

Fig. S6 Polder maps of substrate/product contoured at σ=3.0. UDP-GlcNAc (magenta) in structure B3GNT2_UDPGlcNAc (chain A) **(A)**, LacNAc (orange) in structure B3GNT2_LacNAc (chain A) **(B)**, GlcNAcβ1-3Galβ1-4GlcNAc (teal) **(C)** and UDP (purple) **(D)** in structure B3GNT2_tri_ UDP, and UDP (salmon) in structure B3GNT2_UDP (chain A) **(E)** are shown as sticks. Magnesium ion is shown as a grey sphere.

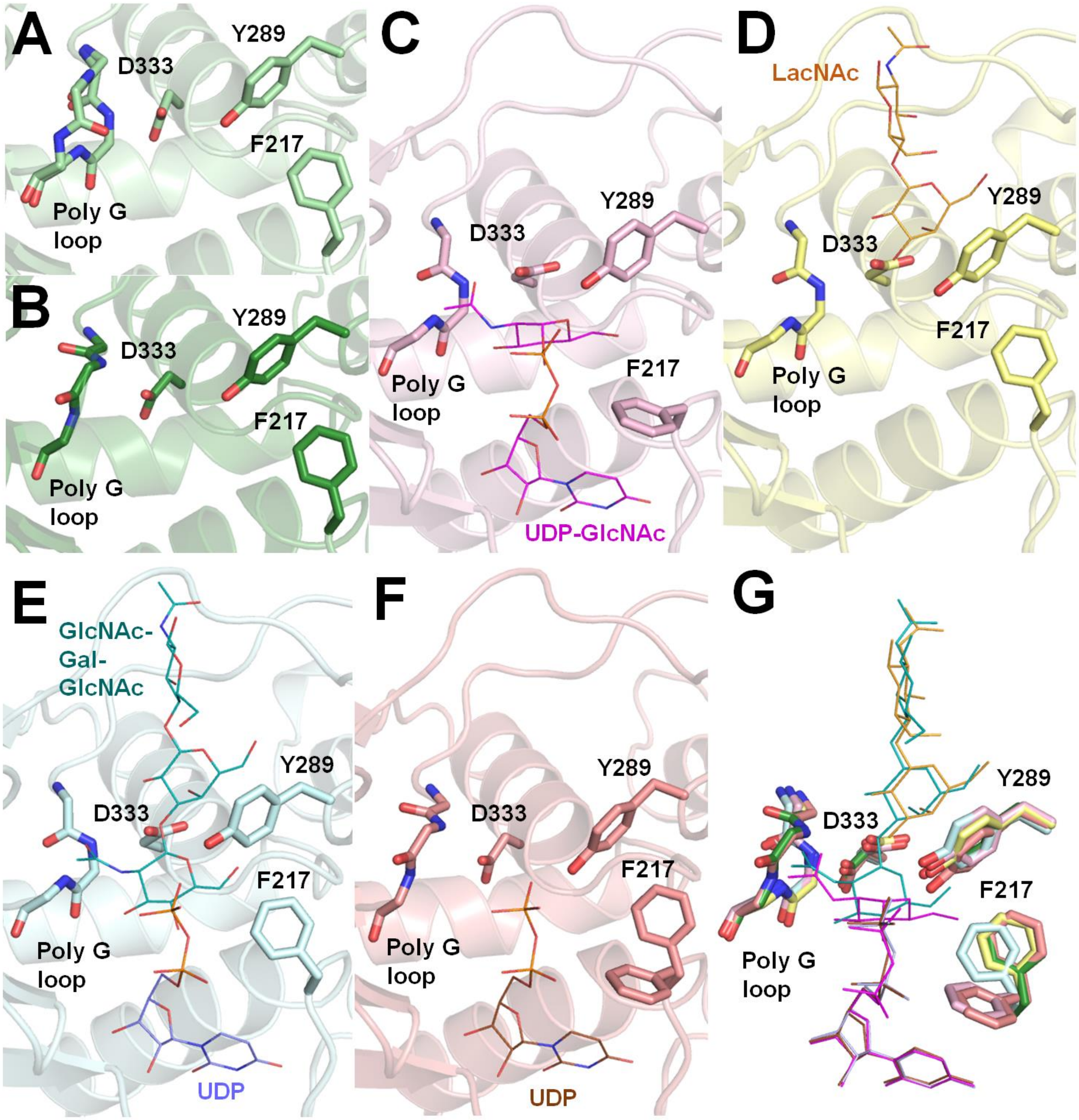

Fig. S7 Conformational changes of the residues in the substrate-binding cleft. The substrate-binding cleft of chain A of B3GNT2_apo **(A)**, chain B of B3GNT2_apo **(B)**, B3GNT2_UDPGlcNAc (chain A) **(C)**, B3GNT2_LacNAc (chain A) **(D)**, B3GNT2_tri_UDP **(E)**, and B3GNT2_UDP (chain A) **(F)**. Residues with conformational changes during the reaction cycle are shown as sticks, including Phe217, Tyr289, Asp333, and the poly Gly loop. Substrate/product in the structures are shown as lines. **(G)** Superposition of B-F.

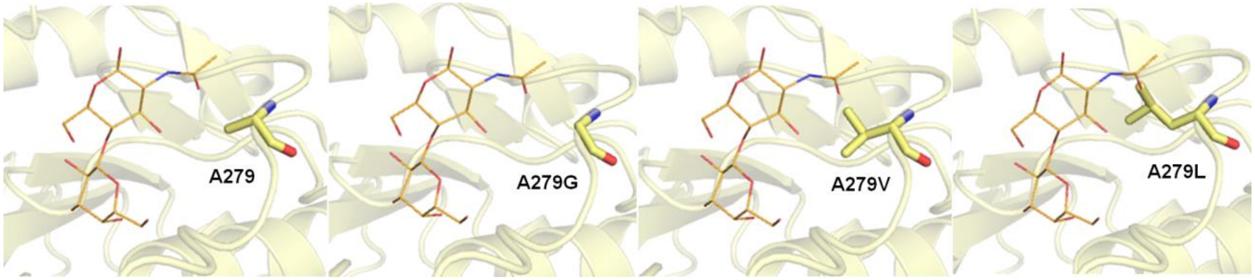

Fig. S8 A simple *in silico* mutagenesis of Ala279. It demonstrates the steric hindrance created at the acceptor substrate binding site in structure B3GNT2_LacNAc (chain A).

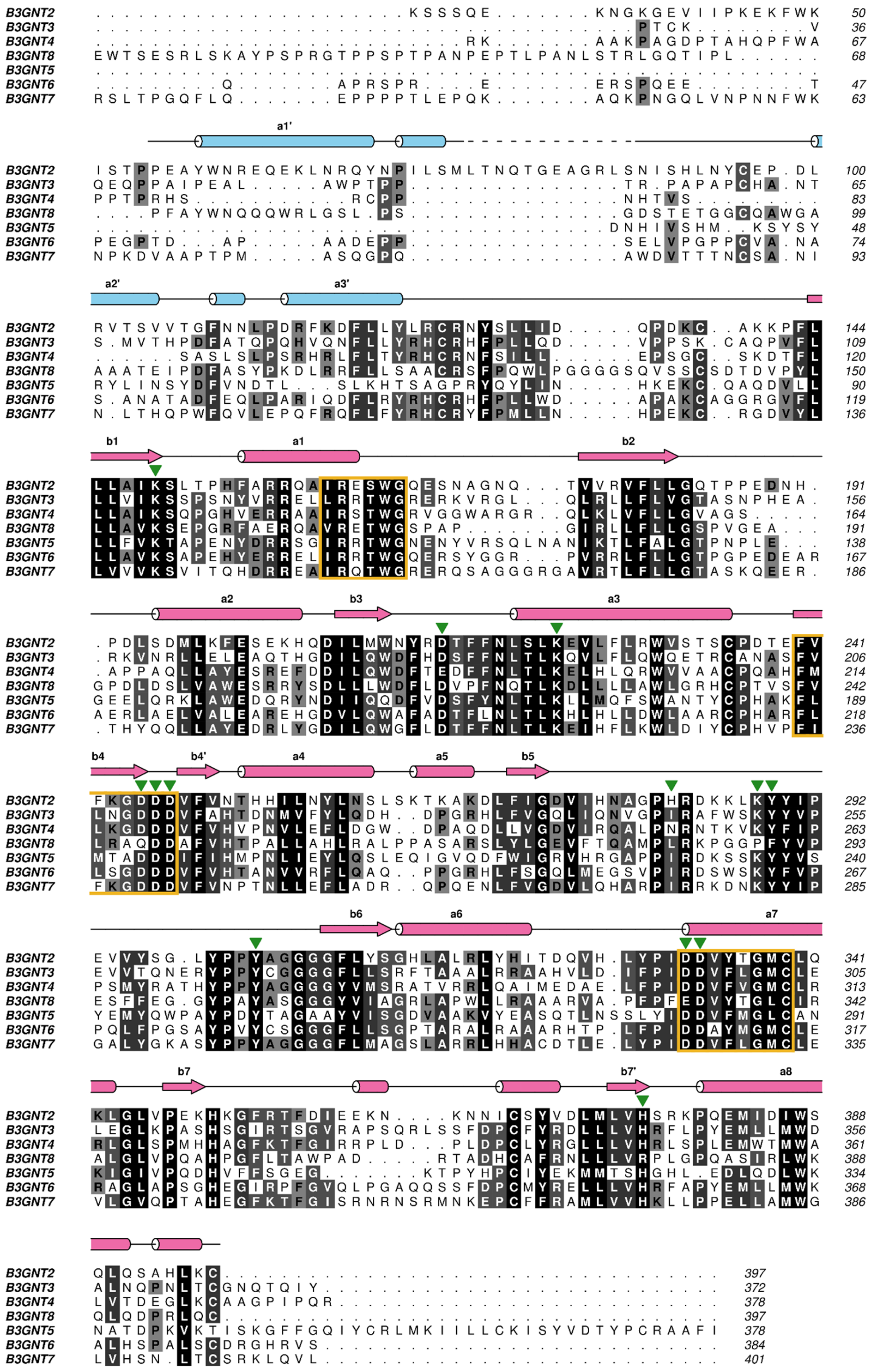

B3GNT2 ....KSSSQE.......KNGKGEVIIPKEKFWK 50
B3GNT3 ....PTCK........V 36
B3GNT4 ....RK.......AAKPAGDPTAHQPFWA 67
B3GNT8 EWTSESRLSKAYPSPRGTPPSPTPANPEPTLPANLSTRLGQTIPL...... 68
B3GNT5 ....
B3GNT6 .........Q.......APRSPR....E.......ERSPQEE........T 47
B3GNT7 RSLTPGQFLQ.......EPPPPTLEPQK.......AQKPNGQLVNPNNFWK 63
a1'
B3GNT2 ISTPPEAYWNREQEKLNRQYNPILSMLTNQTGEAGRLSNISHLNYCEP.DL 100
B3GNT3 QEQPPAIPEAL.....AWPTPP...............TR.PAPAPCHA.NT 65
B3GNT4 PPTPRHS...........RCPP...............NHTVS......... 83
B3GNT8 ....PFAYWNQQQWRLGSL.PS...............GDSTETGGCQAWGA 99
B3GNT5 ....DNHIVSHM..KSYSY 48
B3GNT6 PEGPTD...AP.....AADEPP...............SELVPGPPCVA.NA 74
B3GNT7 NPKDVAAPTPM.....ASQGPQ...............AWDVTTTNCSA.NI 93
a2' a3'
B3GNT2 RVTSVVTGFNNLPDRFKDFLLYLRCRNYSLLID.....QPDKC..AKKPFL 144
B3GNT3 S.MVTHPDFATQPQHVQNFLLYRHCRHFPLLQD.....VPPSK.CAQPVFL 109
B3GNT4 ......SASLSLPSRHRLFLTYRHCRNFSILL......EPSGC..SKDTFL 120
B3GNT8 AAATEIPDFASYPKDLRRFLLSAACRSFPQWLPGGGGSQVSSCSDTDVPYL 150
B3GNT5 RYLINSYDFVNDTL...SLKHTSAGPRYQYLIN.....HKEKC.QAQDVLL 90
B3GNT6 S.ANATADFEQLPARIQDFLRYRHCRHFPLLWD.....APAKCAGGRGVFL 119
B3GNT7 N.LTHQPWFQVLEPQFRQFLFYRHCRYFPMLLN.....HPEKC..RGDVYL 136
b1 a1 b2
B3GNT2 LLAIKSLTPHFARRQAIRESWGQESNAGNQ...TVVRVFLLGQTPPEDNH. 191
B3GNT3 LLVIKSSPSNYVRRELLRRTWGRERKVRGL...QLRLLFLVGTASNPHEA. 156
B3GNT4 LLAIKSQPGHVERRAAIRSTWGRVGGWARGR..QLKLVFLLGVAGS..... 164
B3GNT8 LLAVKSEPGRFAERQAVRETWGSPAP.......GIRLLFLLGSPVGEA... 191
B3GNT5 LLFVKTAPENYDRRSGIRRTWGNENYVRSQLNANIKTLFALGTPNPLE... 138
B3GNT6 LLAVKSAPEHYERRELIRRTWGQERSYGGR...PVRRLFLLGTPGPEDEAR 167
B3GNT7 LVVVKSVITQHDRREAIRQTWGRERQSAGGGRGAVRTLFLLGTASKQEER. 186
a2 b3 a3
B3GNT2 .PDLSDMLKFESEKHQDILMWNYRDTFFNLSLKEVLFLRWVSTSCPDTEFV 241
B3GNT3 .RKVNRLLELEAQTHGDILQWDFHDSFFNLTLKQVLFLQWQETRCANASFV 206
B3GNT4 .APPAQLLAYESREFDDILQWDFTEDFFNLTLKELHLQRWVVAACPQAHFM 214
B3GNT8 GPDLDSLVAWESRRYSDLLLWDFLDVPFNQTLKDLLLAWLGRHCPTVSFV 242
B3GNT5 GEELQRKLAWEDQRYNDIIQQDFVDSFYNLTLKLLMQFSWANTYCPHAKFL 189
B3GNT6 AERLAELVALEAREHGDVLQWAFADTFLNLTLKHLHLLDWLAARCPHARFL 218
B3GNT7 .THYQQLLAYEDRLYGDILQWGFLDTFFNLTLKEIHFLKWLDIYCPHVPFI 236
b4 b4' a4 a5 b5
B3GNT2 FKGDDDVFVNTHHILNYLNSLSKTKAKDLFIGDVIHNAGPHRDKKLKYYIP 292
B3GNT3 LNGDDDVFAHTDNMVFYLQDH..DPGRHLFVGQLIQNVGPIRAFWSKYYVP 255
B3GNT4 LKGDDDVFVHVPNVLEFLDGW..DPAQDLLVGDVIRQALPNRNTKVKYFIP 263
B3GNT8 LRAQDDAFVHTPALLAHLRALPPASARSLYLGEVFTQAMPLRKPGGPFYVP 293
B3GNT5 MTADDDIFIHMPNLIEYLQSLEQIGVQDFWIGRVHRGAPPIRDKSSKYYVS 240
B3GNT6 LSGDDDVFVHTANVVRFLQAQ..PPGRHLFSGQLMEGSVPIRDSWSKYFVP 267
B3GNT7 FKGDDDVFVNPTNLLEFLADR..QPQENLFVGDVLQHARPIRRKDNKYYIP 285
b6 a6 a7
B3GNT2 EVVYSG.LYPPYAGGGGFLYSGHLALRLYHITDQVH.LYPIDDVYTGMCLQ 341
B3GNT3 EVVTQNERYPPYCGGGGFLLSRFTAAALRRAAHVLD.IFPIDDVFLGMCLE 305
B3GNT4 PSMYRATHYPPYAGGGGYVMSRATVRRLQAIMEDAE.LFPIDDVFVGMCLR 313
B3GNT8 ESFFEG.GYPAYASGGGYVIAGRLAPWLLRAAARVA.PFPFEDVYTGLCIR 342
B3GNT5 YEMYQWPAYPDYTAGAAYVISGDVAAKVYEASQTLNSSLYIDDVFMGLCAN 291
B3GNT6 PQLFPGSAYPVYCSGGGFLLSGPTARALRAAARHTP.LFPIDDAYMGMCLE 317
B3GNT7 GALYGKASYPPYAGGGGFLMAGSLARRLHHACDTLE.LYPIDDVFLGMCLE 335
b7 b7' a8
B3GNT2 KLGLVPEKHKGFRTFDIEEKN....KNNICSYVDLMLVHSRKPQEMIDIWS 388
B3GNT3 LEGLKPASHSGIRTSGVRAPSQRLSSFDPCFYRDLLLVHRFLPYEMLLMWD 356
B3GNT4 RLGLSPMHHAGFKTFGIRRPLD...PLDPCLYRGLLLVHRLSPLEMWTMWA 361
B3GNT8 ALGLVPQAHPGFLTAWPAD.....RTADHCAFRNLLLVRPLGPQASIRLWK 388
B3GNT5 KIGIVPQDHVFFSGEG.......KTPYHPCIYEKMMTSHGHL.EDLQDLWK 334
B3GNT6 RAGLAPSGHEGIRPFGVQLPGAQQSSFDPCMYRELLLVHRFAPYEMLLMWK 368
B3GNT7 VLGVQPTAHEGFKTFGISRNRNSRMNKEPCFFRAMLVVHKLLPPELLAMWG 386
B3GNT2 QLQSAHLKC................................... 397
B3GNT3 ALNQPNLTCGNQTQIY............................ 372
B3GNT4 LVTDEGLKCAAGPIPQR........................... 378
B3GNT8 QLQDPRLQC................................... 397
B3GNT5 NATDPKVKTISKGFFGQIYCRLMKIILLCKISYVDTYPCRAAFI 378
B3GNT6 ALHSPALSCDRGHRVS............................ 384
B3GNT7 LVHSN.LTCSRKLQVL............................ 401

Fig. S9 Sequence alignment of the luminal domain of human B3GNT2-B3GNT8. The secondary structure elements of B3GNT2 are shown above the sequence alignment. B3GNT2 active site residues directly involved in substrate/product and metal binding are indicated with green triangles. The three common B3GT sequence motifs are highlighted in yellow boxes. B3GNT1 is not included in the alignment because it is actually a β-1,4-glucuronyltransferase (B4GAT1) [49] instead of a β-1,3-*N*-acetylglucosaminyltransferase. The figure was prepared with ALINE [50].

Table S1 Crystallographic data and refinement statistics

	B3GNT2_I	B3GNT2_apo	B3GNT2_UDPGlcNAc	B3GNT2_LacNAc	B3GNT2_tri_UDP	B3GNT2_UDP
PDB ID	7JHI	7JHK	7JHL	7JHM	7JHN	7JHO
**Data Collection**						
Wavelength (Å)	1.85	1.00	1.00	1.00	1.00	1.00
Space group	P 2_1	P 2_1	I 2	I 2	C 2	C 2
No. of molecules in asymmetric unit	4	4	2	2	1	2
Cell dimensions						
a, b, c (Å)	67.43, 81.39, 157.39	67.42, 80.07, 157.62	82.65, 76.64, 134.21	82.51, 76.99, 134.39	82.92, 76.87, 66.79	137.65, 76.51, 82.70
α, β, γ (°)	90, 98.63, 90	90, 98.82, 90	90, 105.27, 90	90, 105.06, 90	90, 105.82, 90	90, 109.93, 90
Resolution (Å)[a]	47.33-2.50 (2.57-2.50)	100.00-2.34 (2.39-2.34)	46.42-2.26 (2.33-2.36)	46.46-2.19 (2.26-2.19)	50.00-2.20 (2.24-2.20)	30.00-1.85 (1.88-1.85)
R_{merge}	0.131 (1.293)	0.070 (0.437)	0.139 (0.906)	0.101 (0.904)	0.050 (0.506)	0.087 (1.002)
$<I/\sigma(I)>$	17.7 (2.0)	7.4 (2.4)	9.4 (2.4)	11.7 (2.3)	11.2 (1.6)	7.5 (1.5)
Completeness (%)	100.0 (100.0)	95.7 (74.2)	99.9 (100.0)	99.9 (99.9)	86.7 (51.3)	99.8 (97.7)
Multiplicity	13.2 (12.5)	4.6 (3.6)	6.7 (7.0)	6.8 (7.0)	3.5 (2.7)	6.4 (4.9)
**SAD Phasing**						
FOM[b]	0.32					
**Refinement**						
Resolution (Å)	47.33-2.50 (2.54-2.50)	47.18-2.34 (2.38-2.34)	46.42-2.26 (2.32-2.26)	46.46-2.19 (2.24-2.19)	39.98-2.20 (2.37-2.20)	29.22-1.85 (1.88-1.85)
No. of reflections	58414 (2733)	59297 (1680)	37904 (2890)	41864 (2780)	14980 (1325)	64894 (1388)
R_{work}/R_{free}[c]	0.2016/0.2476 (0.2983/0.3572)	0.1792/0.2173 (0.2167/0.3291)	0.1747/0.2241 (0.2308/0.2851)	0.1889/0.2189 (0.2635/0.2877)	0.1955/0.2551 (0.2280/0.3595)	0.1649/0.2111 (0.2428/0.2783)
No. of atoms						
Protein	10812	10879	5502	5484	2712	5625
Ligand/ion[d]	296	305	243	235	143	313
Water	424	516	276	241	107	636
Average B factors (Å^2)						
Protein	30.48	36.97	38.29	41.48	38.25	23.00
Ligand/ion[d]	42.87	58.17	50.85	59.07	56.38	46.04
Water	27.30	39.43	42.44	45.13	34.43	34.94

RMSD						
Bond lengths (Å)	0.003	0.004	0.004	0.004	0.004	0.006
Bond angles (°)	0.592	0.660	0.689	0.782	0.687	0.901
Ramachandran plot (%)						
Favored	96.29	97.70	97.88	97.72	95.05	98.52
Allowed	3.71	2.22	2.12	2.28	4.95	1.48
Outliers	0	0.08	0	0	0	0

[a]The highest-resolution shell is shown in parentheses.

[b]Figure of merit: |F(hkl)best|/|F(hkl)| and calculated before density modification.

[c]$R_{work} = \Sigma_{hkl}(||Fobs_{hkl}| - |Fcalc_{hkl}||)/|Fobs_{hkl}|$, where $|Fobs_{hkl}|$ and $|Fcalc_{hkl}|$ represent the observed and calculated structure factor amplitudes. R_{free} is equivalent to R_{work} but is calculated using 5% of the reflections omitted from refinement.

[d]Ligand/ion in B3GNT2_I: *N*-acetyl-D-glucosamine (NAG), *β*-D-mannopyranose (BMA), *α*-D-mannopyranose (MAN), UDP, magnesium ion, iodide ion, chloride ion, tetraethylene glycol. Ligand/ion in B3GNT2_apo: NAG, BMA, MAN, sodium ion, chloride ion. Ligand/ion in B3GNT2_UDPGlcNAc: NAG, BMA, MAN, UDP-GlcNAc, magnesium ion, chloride ion, triethylene glycol. Ligand/ion in B3GNT2_LacNAc: NAG, BMA, MAN, LacNAc, hexaethylene glycol. Ligand/ion in B3GNT2_tri_UDP: NAG, BMA, MAN, GlcNAc*β*1-3Gal*β*1-4GlcNAc, UDP, magnesium ion. Ligand/ion in B3GNT2_UDP: NAG, BMA, MAN, *α*-L-fucose (FUC), UDP, magnesium ion, chloride ion, glycerol.

Table S2 Primers used for *B3GNT2* mutagenesis

	Forward	Reverse
H376L	tcatctcttgaggttttctactaagtactaacatcagatctacatag	ctatgtagatctgatgttagtacttagtagaaaacctcaagagatga
H376E	tcatctcttgaggttttctactctctactaacatcagatctacataggag	ctcctatgtagatctgatgttagtagagagtagaaaacctcaagagatga
H376Q	cttgaggttttctactctgtactaacatcagatctacataggagc	gctcctatgtagatctgatgttagtacagagtagaaaacctcaag
D247A	gggtgttcacaaaaacagcgtcatcgcccttgaaa	tttcaagggcgatgacgctgtttttgtgaacaccc
D333N	ggtccatctctaccccattgataacgtttatactgga	tccagtataaacgttatcaatggggtagagatggacc
A279G	atcccgatgaggtccaccattgtggatcacatc	gatgtgatccacaatggtggacctcatcgggat
A279V	tatcccgatgaggtccaacattgtggatcacatca	tgatgtgatccacaatgttggacctcatcgggata
A279L	tcccgatgaggtcctagattgtggatcacatcacctatgaagag	ctcttcataggtgatgtgatccacaatctaggacctcatcggga
D245A	gttcacaaaaacatcgtcagcgcccttgaaaacaaactc	gagtttgttttcaagggcgctgacgatgtttttgtgaac
D332A	cattccagtataaacgtcagcaatggggtagagatggac	gtccatctctaccccattgctgacgtttatactggaatg
K149A	gtggagtgagggacgcaatcgccagcaacaagaaaggtttct	agaaacctttcttgttgctggcgattgcgtccctcactccac
Y289F	acaacttctgggatgtagaacttcagcttcttatccc	gggataagaagctgaagttctacatcccagaagttgt

References

49. Praissman, J.L., Live, D.H., Wang, S., Ramiah, A., Chinoy, Z.S., Boons, G.J., Moremen, K.W., and Wells, L., *B4GAT1 is the priming enzyme for the LARGE-dependent functional glycosylation of alpha-dystroglycan*. Elife, 2014. **3**: e03943. doi: 10.7554/eLife.03943.
50. Bond, C.S. and Schuttelkopf, A.W., *ALINE: a WYSIWYG protein-sequence alignment editor for publication-quality alignments.* Acta Crystallogr D Biol Crystallogr, 2009. **65**(Pt 5): p. 510-2.
